# Engineering of Industrial Kraft Lignin: The Role of Esterification Methods in Lignin Nanoparticle Self-Assembly

**DOI:** 10.1021/acs.biomac.5c00507

**Published:** 2025-08-18

**Authors:** Taoran Xu, Anastasia V. Riazanova, Pär A. Lindén, Gunnar Henriksson, L. Daniel Söderberg, Oihana Gordobil, Olena Sevastyanova

**Affiliations:** † Wallenberg Wood Science Center, Department of Fiber and Polymer Technology, 298098 KTH Royal Institute of Technology, School of Chemistry, Biotechnology and Health, Teknikringen 56, Stockholm, 100 44, Sweden; ‡ Department of Fiber and Polymer Technology, School of Chemistry, Biotechnology and Health, Teknikringen 56, Stockholm, 100 44, Sweden; § “Materials + Technologies” Research Group (GMT), Department of Chemical and Environmental Engineering, University of the Basque Country UPV/EHU, Faculty of Engineering of Gipuzkoa, Plaza Europa 1, Donostia-San Sebastián 20018, Spain

## Abstract

Lignin nanoparticles (LNPs) are gaining increasing interest for applications in various fields, where the particle homogeneity, morphology, and surface properties are critical for performance. In this study, lignin obtained via kraft process from spruce and eucalyptus was employed as precursor for the fabrication of lignin nanoparticles with tunable physicochemical properties. Linear ester groups with varying chain lengths were introduced to systematically investigate the effects of the hydrophobic moiety distribution on lignin nanoparticle formation via solvent-shifting self-assembly. Results demonstrated that esterification-induced structural changes altered the balance of key noncovalent interactions (hydrogen bonding, π–π stacking, and hydrophobic interactions), which collectively governed the self-assembly process, with longer ester chains promoting compact particles with hydrophobic surfaces. By directly linking molecular-level modification of lignin to alterations in the inter- and intramolecular interactions driving the self-assembly of nanoparticles, this study provides a mechanistic framework for the rational design of lignin nanoparticles through controlled chemical modification, thereby expanding their application flexibility.

## Graphical abstract

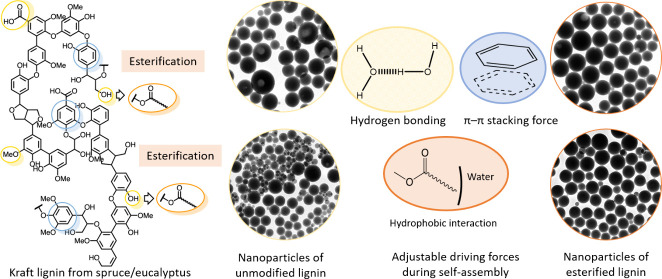

## Introduction

The exploration of biobased and sustainable alternatives to traditional petroleum-based products, with a focus on circularity, is being actively pursued through ongoing research into the use of industrial byproducts as raw materials.^1^ Lignin, a naturally abundant aromatic polymer generated from chemical pulping of wood and other plant materials, stands out as one of the alternatives.^2^ This biopolymer is chemically complex and heterogeneous and is biosynthesized through the free radical coupling of mainly three major precursor monolignols with varying methoxylation on their aromatic rings: *p*-hydroxyphenyl (H), guaiacyl (G), and syringyl (S) units. These units are linked by carbon–carbon (β-1, β-5, β-β, 5-5) and ether (β-O-4, α-O-4, 4-O-5) bonds. Lignin contains several functional groups, primarily aliphatic/phenolic hydroxyl groups, carboxyl groups and methoxy groups.^3^ The kraft pulping method involves hydrolysis of α-ethers and cleavage of β-aryl ether linkages, demethylation reaction, formation of vinyl ethers and stilbenes, and condensation reaction at the C_5_ position in the aromatic ring.^4,5^ These reactions generate macromolecules with rather large differences from natural lignin with increased phenolic functionality, decreased average molecular weight, and increased polydispersity, enhancing the solubility of lignin in alkaline and some nonpolar organic solvents. The biosynthetic pathways, degree of cross-linking and relative amounts of the precursors vary with the bioresources of lignin, and structural changes in lignin depend heavily on the industrial extraction processes, contributing to its physicochemical heterogeneity.^6^ Due to its chemical and biological properties, such as renewability, biodegradability, and partial biocompatibility, lignin valorization becomes an essential process for sustainable biorefineries.^7^ However, only a small amount of kraft lignin is applied in low-value industrial applications due to its physicochemical heterogeneity and the lack of precise knowledge about its real structure.^8^

The hydroxyl groups in lignin exhibit high reactivity toward nucleophilic substitution, allowing complex functionalization such as esterification, phenolation, etherification and urethanization,^9^ making lignin a valuable precursor in further chemical processes.^10^ Esterification is considered one of the easiest reactions to carry out, considering the reaction parameters and reactant used. Various esterification methods have been employed to improve the thermal and mechanical properties of lignin-based copolymers,^11^ to improve the compatibility of lignin with polymetric matrix thus to improve physiochemical properties of the composites,^12^ or to introduce new chemically reactive sites into lignin.^13^

Fabricating lignin into functional nanomaterials, particularly into lignin nanoparticles (LNPs), has garnered significant attention over the past decades.^14^ The unique properties of lignin nanoparticles allow them to be used in various application fields, including cosmetics (antioxidants, anti-UV products^15^), medical care (delivery systems for anticancer-drugs^16^), agriculture (controlled release of fertilizers^17^ or pesticides^18,19^ ), pollution treatment (absorbents for pigments^20^ and coagulants for heavy metals^21^), energy generation (electrodes^22^ and binders^23^ for battery units), composite materials (reinforcements for resins^24^ and plastic replacement materials^25^), and construction materials (fillers of cement^26^). The controllable production of lignin nanoparticles (LNPs) involves various chemical, physical and biological approaches, including solvent shifting,^27^ ultrasonication,^28^ interfacial cross-linking,^29^ surface grafting,^30^ and enzyme-aided assembly.^31^

The most conventional and intensively studied methods are solution-based processes that follow the principles of self-assembly, which stand out due to relatively low costs, high yield of LNPs and good control over LNPs morphology and functionalities.^32,33^ However, the field of lignin nanotechnology is still in its early stages. The main challenges in this field are related to the high heterogeneity and chemical variability of lignin polymer, lack of understanding of lignin macromolecule behavior during LNPs formation, and the nanostructure–properties relationships and interaction mechanisms. Limitations, such as the toxicity or difficult recovery of some organic solvents, also exist.^34^

The inter- and intramolecular interactions within lignin macromolecules and solvent molecules are considered the essential driving forces throughout the spontaneous formation of LNPs. The interactions systematically categorized into moderate to strong polar forces (electrostatic interactions and hydrogen bonding) and nonpolar forces (π–π stacking and van der Waals forces), being responsible for various types of aggregations phenomena during lignin nanoparticle self-assembly.^35,36^ Different lignin domains bearing varying functional moieties tend to reorganize their secondary conformations as a result of these collaborative interactions and eventually aggregate into nanoparticles.^37^

Lignin nanoparticle self-assembly via binary solvent-shifting can be considered as two stages of gradual aggregation. Lignin is normally dissolved in a water-miscible organic solvent first, followed by direct addition of antisolvent or by dialysis within antisolvent. The first stage is the primary nucleation to form colloidal clusters to effectively minimize the contact with polar solvent phase and to minimize the surface energy, where short-range interactions are dominating: hydrogen bonds (by phenolated, hydroxylated, and carboxylated moieties) and nonpolar π–π interactions (by aromatic skeletons) are strengthened during the addition of antisolvent, leading most hydrophobic domains to collide.^33,38–40^ The second stage is the growth and maturing of lignin nanoparticles by gradual aggregation of less hydrophobic domains onto the cluster surface, being governed by long-range interactions, particularly van der Waals and hydrophobic interactions (by entanglement of phenyl propanoid alkylated chains and other nonpolarizable functional moieties).^41–43^ Studies have shown that hydrophobic forces, in response to the polarity shift of solvent systems, play a critical role in reorganizing lignin domains into spherical, compact and even colloidal cores.^29,44,45^

Some studies have explored modifying the hydrophobic/hydrophilic balance of lignin by acetylation,^46,47^ methylation,^48^ maleation,^49^ methacrylation,^50^ oleation,^37^ etc. Such lignin with increase hydrophobicity could be utilized to improved thermal/mechanical properties in nanocomposites such as controlled-release pesticides,^19^ environmentally friendly surfactants, and hydrophobic coatings.^51^ However, these works exclusively focused on application-driven properties of material composites, such as improved thermal stability, hydrophobicity, and compatibility with other materials. Addressing a fundamental knowledge gap of how tailored hydrophobic/hydrophilic modifications systematically affect the self-assembly pathway and ultimate nanoparticle architectures could help refine lignin nanoparticle production protocols and expand their applications.

This strategy offers an experimentally grounded framework for reevaluating how lignin structure influences LNP formation and properties, with particular attention to subtle yet critical physicochemical variations introduced through chemical modification. This study focuses on (i) systematically varying lignin’s hydrophobicity through esterification with linear aliphatic chains of different lengths; (ii) quantifying the effect of resulting lignin hydrophobic/hydrophilic balance on LNP size and surface properties; (iii) demonstrating the cooperation of hydrophobic interactions, hydrogen bonding, and π–π stacking forces in LNP assembly control. A systematic investigation in how esterification of lignin impacts their self-assembly events during binary solvent-shifting processes is performed, with each esterified lignin variant serving as a controlled reference against the others, enabling a direct comparison of the key intra- and intermolecular forces that drive LNP nucleation and maturation. The research findings provide a more comprehensive understanding of how the introduction of various hydrophobic moieties influences the physicochemical behaviors of lignin nanoparticles, ultimately guiding their design for targeted applications.

## Experimental Methods

### Materials

Ethanol, acetone, pyridine, *N*,*N*-dimethylformamide, triethylamine, acetic acid anhydride, hexanoyl chloride, dodecanoyl chloride, hydrochloride acid, deuterated dimethyl sulfoxide, deuterated chloroform, deuterated acetone, deuterium oxide, 2-chloro-4,4,5,5-tetramethyl-1,3,2-dixoaphosphlane, *N*-hydroxy-5-norbornene-2,3dicarboximide, and chromium­(III) pentanedionate were purchased from Sigma-Aldrich (Stockholm, Sweden). LignoBoost spruce kraft lignin (SKL) and LignoBoost eucalyptus kraft lignin (EKL) were kindly provided by Stora Enso (Stockholm, Sweden) and Suzano (São Paulo, Finland), respectively.

### Lignin Chemical Modification

#### C2-Esterification of Lignin Samples

The acetylation of lignin was performed according to Gordobil et al.^47^ For this, 1 g of dry kraft lignin from Norway spruce and eucalyptus was dispersed in 30 mL of pyridine until complete solubilization. Acetic acid anhydride (1.1 mol per total hydroxyl group) was added in 3 equal portions at 3 h intervals after the addition of pyridine. The reaction was maintained under vigorous stirring (800 rpm) with a magnetic stirrer bar for 30 h. The resulting solution was poured into 500 mL of 0.1 N hydrochloric acid and allowed to precipitate in an ice bath for 30 min. The precipitate was collected by using a 0.45 μm nylon filter and washed with excess ethanol and distilled water. The precipitate was further rinsed with excess ethanol three times and dried on the filter at room temperature for 48 h.

#### C6- and C12-Esterification of Lignin Samples

The esterification of lignin was performed according to Gordobil et al.^52^ For this, 1 g of dry kraft lignin from Norway spruce or eucalyptus was dispersed in 30 mL of N, N-dimethylformamide until complete solubilization. Pyridine (5.5 mL) was used as a catalyst, and triethylamine (1.5 mL) was added to trap the chloride acid formed during the reaction. Exceed amounts of hexanoyl chloride and dodecanoyl chloride (1.1 mol per total hydroxyl group) were added, respectively. The reaction was maintained under vigorous stirring (800 rpm) with a magnetic stir bar for 4 h. The resulting solution was poured into 650 mL of 0.1 N hydrochloride acid and allowed to precipitate in an ice bath for 30 min. The precipitate was collected using a 0.45 μm nylon filter and washed with excess distilled water and absolute ethanol. The precipitate was rinsed with excess ethanol 3 times and dried on the filter at room temperature for 48 h. The reaction principles of esterification are illustrated in Scheme 1.

**Scheme 1:**
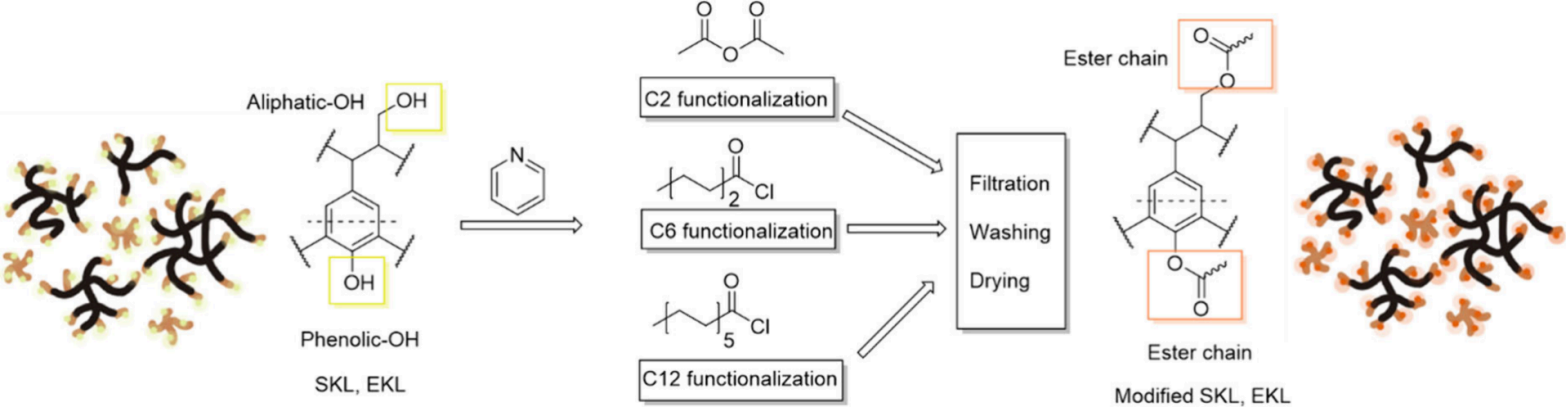
Esterification of Kraft Lignin

### Lignin Nanoparticle Fabrication

The original and esterified lignin samples were dissolved in acetone and filtered through a 0.45 μm membrane filter to remove undissolved material and possible aggregates, yielding a solution with a lignin concentration of 10 mg/mL lignin. Under moderate stirring (300 rpm) using a 1 cm magnetic stirrer bar, deionized water was added dropwise (0.2 mL/min) to the lignin solution at a volume ratio of 4:1 (water to lignin solution) in a 10 mL cylindrical glass vial (2 mL in diameter) to induce nanoprecipitation.^35^ The solution was stirred for 48 h to allow acetone to evaporate, resulting in a lignin nanoparticle suspension. The obtained lignin nanoparticle suspension was transferred into a dialysis bag (3.5 kDa molecular weight cutoff) and dialyzed against deionized water for 72 h to effectively remove residue acetone. The final concentration of lignin in the lignin nanoparticle suspension was 2.5 mg/mL. The self-assembly procedures are illustrated in Scheme 2.

**Scheme 2:**
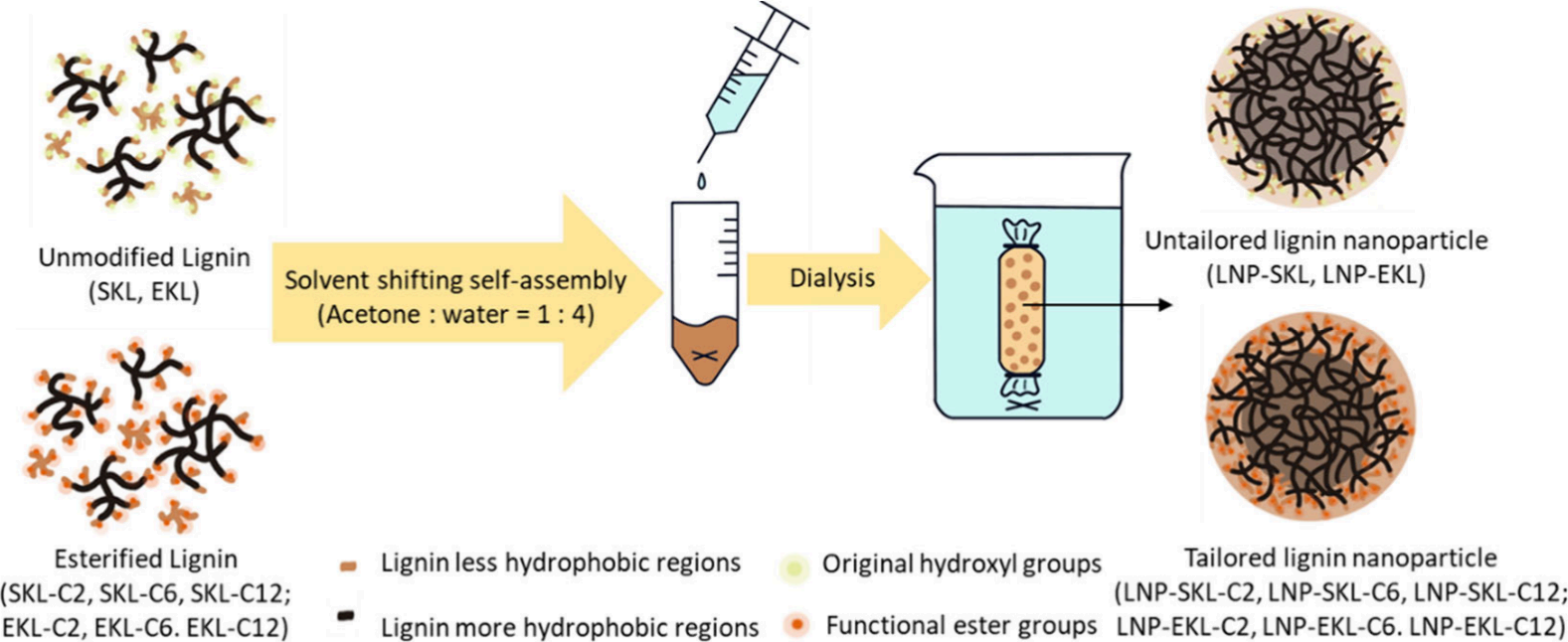
Self-Assembly of Unmodified/Modified Kraft Lignin Nanoparticles in Acetone/Water Binary System

## Characterization

### Fourier Transform Infrared Spectroscopy (FTIR)

Fourier transform infrared spectroscopy (FTIR) was performed to analyze the chemical structural differences in lignin samples. Lignin samples were dried under room temperature for 24 h before being pressed into thin sheets of uniform thickness and measured using PerkinElmer Frontier spectrum 2000 FTIR. The spectra were collected under a wavenumber range of 4000 to 600 cm^–1^, with a resolution of 2 cm^–1^ using 16 scans, and proceeded by PerkinElmer Spectrum software with baseline correction and normalization.

### Size Exclusion Chromatography (SEC)

The weight-average molecular weight (*M*_w_), the number-average molecular weight (*M*_n_) and the polydispersity index (PDI), of the lignin samples were determined by the SEC method according to Guerra et al.^53^ Samples were acetobrominated prior to analysis: 5 mg of sample was stirred for 2 h at room temperature in 1 mL of 9/1 (v/v) mixture of glacial acetic acid and neat acetyl bromide before surplus reagents were removed rapidly *in vacuo*. The residue was dissolved in 1 mL of HPLC grade tetrahydrofuran (THF), and the resulting solution was filtered through a 5 μm syringe filter. SEC analysis was performed using a Waters instrument system (Waters Sverige AB, Sollentuna, Sweden) consisting of a 515 HPLC-pump, 2707 autosampler and 2998 photodiode array detector (operated at 254 and 280 nm). HPLC-grade tetrahydrofuran, filtered through a 2 μm PTFE membrane filter and degassed, was used as a mobile phase using a flow of 0.3 mL/min. Separation was achieved on Waters Ultrastyragel HR4, HR2 and HR0.5 4.6 × 300 mm solvent efficient columns connected in series and operated at 35 °C. For analysis, a sample volume of 20 μL was injected using the partial loop needle overfill injection technique. Data was collected at both 254 and 280 nm to ensure minimal peak drift. Calibration was performed at 254 nm using polystyrene standards with nominal molecular weights ranging from 480 to 176000 Da. Quantification was performed using the Waters Empower3 build 3471 software suite.

### Heteronuclear Single Quantum Coherence Nuclear Magnetic Resonance Spectroscopy (HSQC NMR)

HSQC NMR (^13^C^1^H correlated) was performed to check the skeleton changes of the lignin samples. Lignin samples were prepared by dissolving 80 mg of sample in 600 μL of deuterated dimethyl sulfoxide (DMSO-*d*_6_) respectively. The HSQC NMR data were acquired on a Bruker NMR spectrometer Advanced III HD 400 MHz, using the “hsqcetgpsi” pulse program using 86 scans (and 16 additional dummy scans) over 1024 × 256 increments, a relaxation delay of 1.5 s, an acquisition time of 0.087 s, and a spectral window of 166.7 ppm on F1 and 14.7 ppm on F2. The data was processed in MestReNova (version 9.0.0, Mestrelab Research) using 1024 × 1024 data points using a 90°-shifted square sine-bell apodization window. The data was Fourier transformed followed by phase correction and baseline correction which was applied in both dimensions by means of a Bernstein polynomial fit of order 3.^54^

### Phosphorus-31 Nuclear Magnetic Resonance Spectroscopy (^31^P NMR)

Phosphorus-31 nuclear magnetic resonance spectroscopy was carried out to determine the hydroxyl content of the lignin samples. A 30 mg (accurately weighed) sample of SKL, EKL, SKL-C2, and EKL-C2, respectively, was phosphorylated with 50 μL of 2-chloro-4,4,5,5-tetramethyl-1,3,2-dixoaphosphlane (TMDP) in 650 μL of pyridine/DMF/CDCl_3_ (1:1:4.5, v/v/v) containing *N*-hydroxy-5-norbornene-2,3-dicarboximide (60 mg/mL) as the internal standard and chromium­(III) pentanedionate (5 mg/mL) as the relaxation reagent; A 10 mg sample of SKL-C6, EKL-C6, SKL-C12 and EKL-C12, respectively, was phosphorylated with 20 μL of 2-chloro-4,4,5,5-tetramethyl-1,3,2-dixoaphosphlane (TMDP) in 350 μL of pyridine/DMF/CDCl_3_ (1:1:4.5, v/v/v) containing *N*-hydroxy-5-norbornene-2,3-dicarboximide (60 mg/mL) and chromium­(III) pentanedionate (5 mg/mL) as the internal standard and transferred to a 5 mm NMR tube after 1 h of reaction. The quantitative ^31^P NMR data were acquired on a Bruker Advanced III HD 400 MHz NMR spectrometer using a 5 mm triple resonance probe. A 90° pulse width, 1.2 s acquisition time, and 5 s pulse delay were used in collecting 256 scans.^55^ The data was processed in MestReNova (version 9.0.0, Mestrelab Research)

Given the assumption of preserved lignin backbone after esterification, the relative hydroxyl group contents (mol_–OH group_/mol_lignin sample_) were calculated as

$$-\mathrm{OH group molar content}=-\mathrm{OH group weight relative content}\times M_{n\mathrm{Lignin sample}}$$

The conversion degree of hydroxyl groups in percentage was calculated as

$${\mathrm{conversion degree}}_{-\mathrm{OH group}}=\left[({-\mathrm{OH group molar content}}_{\mathrm{unmodified sample}}-{-\mathrm{OH group molar content}}_{\mathrm{esterified sample}})/{-\mathrm{OH group molar content}}_{\mathrm{unmodified sample}}\right]\times 100\%$$

The estimated loading of ester groups (mol_ester group_/mol_lignin sample_) was calculated as

$$-\mathrm{Loaded ester group molar ratio}=\left\{\left[\left({-\mathrm{OH group molar content}}_{\mathrm{unmodified sample}}-{-\mathrm{OH group molar content}}_{\mathrm{esterified sample}}\right)\times M_{n\mathrm{Ester group}}\right]/\left({-\mathrm{OH group molar content}}_{\mathrm{unmodified sample}}\times M_{n\mathrm{Lignin sample}}\right)\right\}$$

### Transmission Electron Microscopy (TEM)

Transmission electron microscopy was used to observe the morphology of the lignin nanoparticles. TEM analysis was performed on a Hitachi HT7700 series instrument (Hitachi, Japan), at an accelerating voltage of 100.0 kV and an emission current of 8.0 μA. Samples were prepared in the following way: a total of 5 μL of the obtained lignin nanoparticle suspension was drop-cast on a 200-mesh copper grid (Ted Pella Inc.; prod no. 01800-F) and dried in air for 30 min.^56^

### Hydrogen-1 Nuclear Magnetic Resonance Spectroscopy (^1^H NMR)

^1^H NMR was done to characterize the surficial chemical structure of lignin nanoparticles in suspension, according to the method developed by Pylypchuk et al.^57^ Lignin nanoparticles were produced by identical methods with acetone-*d*_6_ and deuterium oxide: The original and chemically modified lignin samples were dissolved in acetone and filtered through a 0.45 μm membrane filter to remove undissolved material and possible aggregates to give a solution with a concentration of 10 mg/mL lignin. Under moderate stirring, 4 volumes of deionized water was then added dropwise to a lignin solution for nanoprecipitation. The solution was stirred for 2 days to evaporate acetone-*d*_6_ and to give a lignin nanoparticle deuterium oxide suspension of 2.5 mg/mL. The according suspensions were diluted 3 times with deuterium oxide and sonicated for 1 min to avoid aggregation. All NMR experiments were carried out on a Bruker AvanceIII 400 instrument (Bruker Corporation, Billerica, MA) equipped with a 5 mm Bruker BBO probe (Bruker Corporation, Billerica, MA). The number of scans of each experiment was 4096, with a relaxation delay time of 5.5 s and an acquisition time of 2.55 s. All NMR data were collected and processed using MestReNova (v.9.0.0, Mestrelab Research).

### Dynamic Light Scattering (DLS)

Dynamic light scattering was performed to measure the hydrodynamic diameter and zeta potential of the lignin nanoparticles in suspension. All obtained lignin nanoparticle suspensions were diluted 20 times and presonicated to give nearly colorless and transparent solutions, which were subsequently analyzed using Zetasizer Nano ZS instrument (Malvern-Pananalytical, Malvern, UK). All measurements were performed in at least triplicate, by taking three different portions of diluted suspensions, with 60 s of equilibrium time at 25 °C. The measurements were performed at 173° in the backscattering mode.

### Contact Angle Measurement (CA)

Contact angles of the lignin nanoparticle suspension droplets on a silicon wafer were measured to compare the wetting properties of lignin nanoparticles in a water suspension. The obtained lignin nanoparticle suspensions were presonicated for uniform dispersion. A silicon wafer was used as a substrate, which was cleaned using sequential rinsing with acetone, ethanol, and deionized water, followed by drying under a nitrogen stream. Five μL droplets of each suspension were deposited onto the silicon wafer using a micropipette. The droplets were allowed to stabilize for 30 s before measurement. The static contact angle was recorded using a OneAttension Optical Tensiometer equipped with an optical camera, and 50 measurements were taken for statistical analysis.

## Results and Discussion

### Lignin Esterification

#### Qualification of Successful Esterification

The FTIR spectra (seen in Figure 1) confirmed the substitution of hydroxyl groups to various ester chains through esterification, as evidenced by a notable decrease in the intensity of the O–H stretching signals from hydroxyl groups (around 3440 cm^–1^). This was accompanied by a significant increase in C=O stretching signals (around 1760 and 1740 cm^–1^, corresponding to the aromatic and aliphatic ester bonds, respectively) and C–H stretching signals (around 2940 and 2832 cm^–1^, attributed to alkyl groups), both characteristic of ester chains.

**Figure 1:**
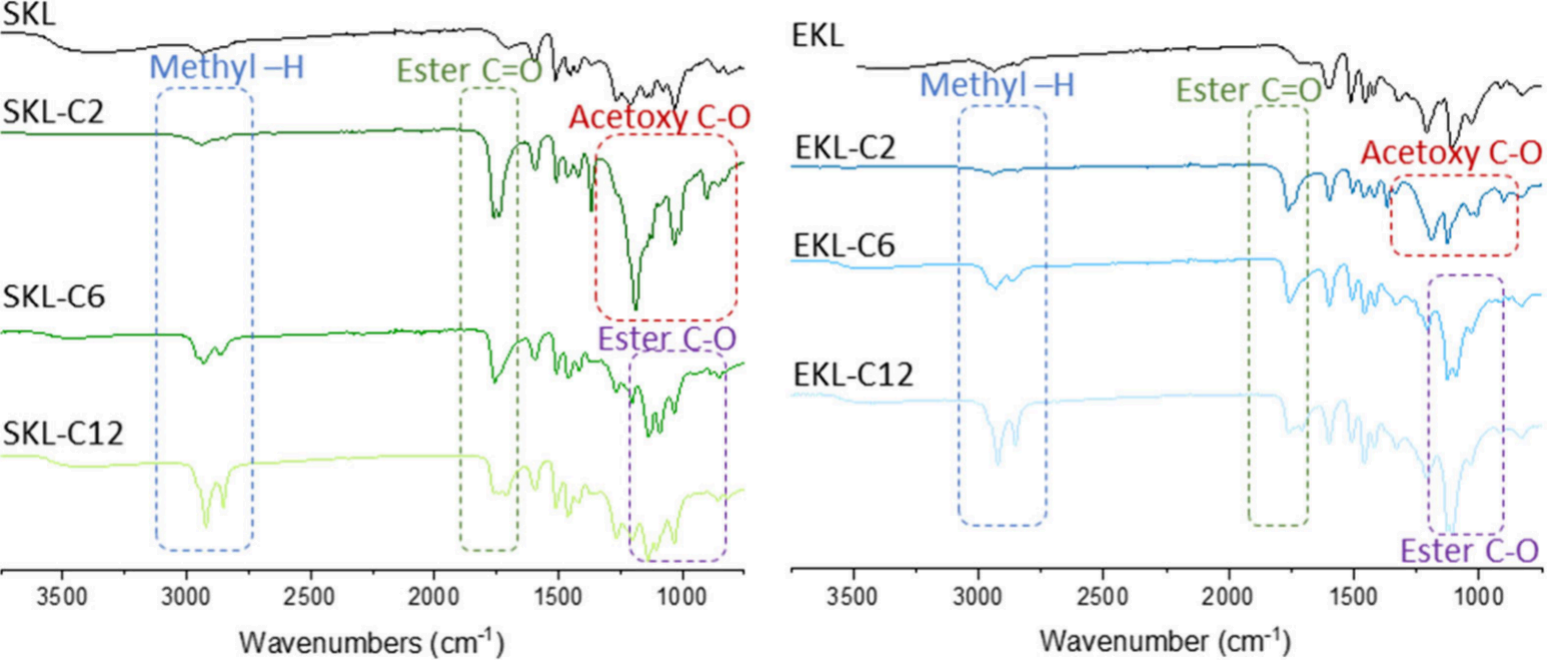
FTIR spectra of SKL and EKL derivatives.

For the C2-esterified samples, an increase in C–O stretching signals (around 1100 and 1030 cm^–1^) indicated the incorporation of acetyl groups; For the C6- and C12-esterified samples, an increase in C–O stretching signals (around 1100 to 1300 cm^–1^) indicated the attachment of hexanoyl and dodecanoyl ester groups. Additionally, variations in the intensity of the C–H stretching signal correlated with differences in the ester chain lengths of each esterified sample. More detailed peak assignments can be found in Supporting Information (Table S1).

HSQC analysis revealed the presence of H, G, and S units, interunit linkages, and successful esterification of SKL-C2, SKL-C6, SKL-C12, EKL-C2, EKL-C6, and EKL-C12. Figures S1 and S2 show HSQC spectral regions revealing structural details (peak assignments provided in Tables S2 and S3). Signals for G units, H units, and acetylated G units were found in SKL and derivatives; Signals corresponding to G units, S units and acetylated G and S units were observed in EKL and derivatives. The relative abundances of β-O-4′ and β-β′ linkages in the aromatic region, which are characteristic of the lignin backbone integrity, were evaluated by calculating the ratio of their respective peak integrations (shown in Table S4). The β-O-4′/β-β′ abundance ratios of SKL and EKL were 2.73 and 3.03, respectively, the ratios showed a very minor decrease (within 5%) after several esterification processes: A small fraction of lignin with low molecular weight was likely washed away by the involvement of organic solvents during modification, and such workup would lead to disproportional changes in SEC measurements.^58,59^ The following discussions are based on the assumption that no significant disruption of the major aromatic framework happened during esterification in order to simplify the comparison of sample properties.

#### Identification of Lignin Macromolecule Structural Changes

According to ^31^P NMR analysis (spectra shown in Figure S4), the loading of ester groups was found to depend on the substructure and abundance of hydroxyl groups, suggesting a different reactivity between phenolic and aliphatic hydroxyl groups. The hydroxyl group contents (mmol/g of lignin) in both unmodified and esterified lignin samples are listed in Supporting Information (Table S5) and the corresponding hydroxyl groups conversion degrees are presented in Table 1.

**Table 1:** Conversion of Hydroxyl Groups in Percentage

sample	conversion of aliphatic–OH (%)	conversion of phenolic–OH (%)	conversion of total −OH
SKL			
SKL-C2	93	93	93
SKL-C6	24	71	55
SKL-C12	6	19	15
EKL			
EKL-C2	88	93	92
EKL-C6	21	62	51
EKL-C12	6	18	14

The esterification efficiency decreased with increasing ester chain length, as longer ester groups exhibited greater steric hindrance, impeding their attachment to lignin hydroxyl groups. This trend was evidenced by the reduced hydroxyl group conversion degree observed with increasing ester chain length.^60^ Additionally, with the longer chain length in the base-catalyzed systems, phenolic hydroxyls demonstrated higher reactivity toward esterification compared to aliphatic hydroxyls, likely attributed to differences in the nucleophilicity of their reaction intermediates.^61–63^ There was no striking differences in conversion degree of either aliphatic or phenolic hydroxyl groups in spruce and eucalyptus kraft lignin, likely due to their moderate reaction kinetics: the efficiency appeared to dependent primarily on the same exceeded stoichiometric ratio of the reagents.^64,65^ The total hydroxyl conversion degrees of both lignin samples in the same reaction conditions were comparable, likely governed by the reaction of their more abundant phenolic groups.

Figure 2 and Table S5 show the calibrated results of SEC analysis. The molecular structural changes of lignin upon C2, C6, and C12 esterification (average molecular weight changes and estimated ester group loading) were compared in Table 2.

**Figure 2:**
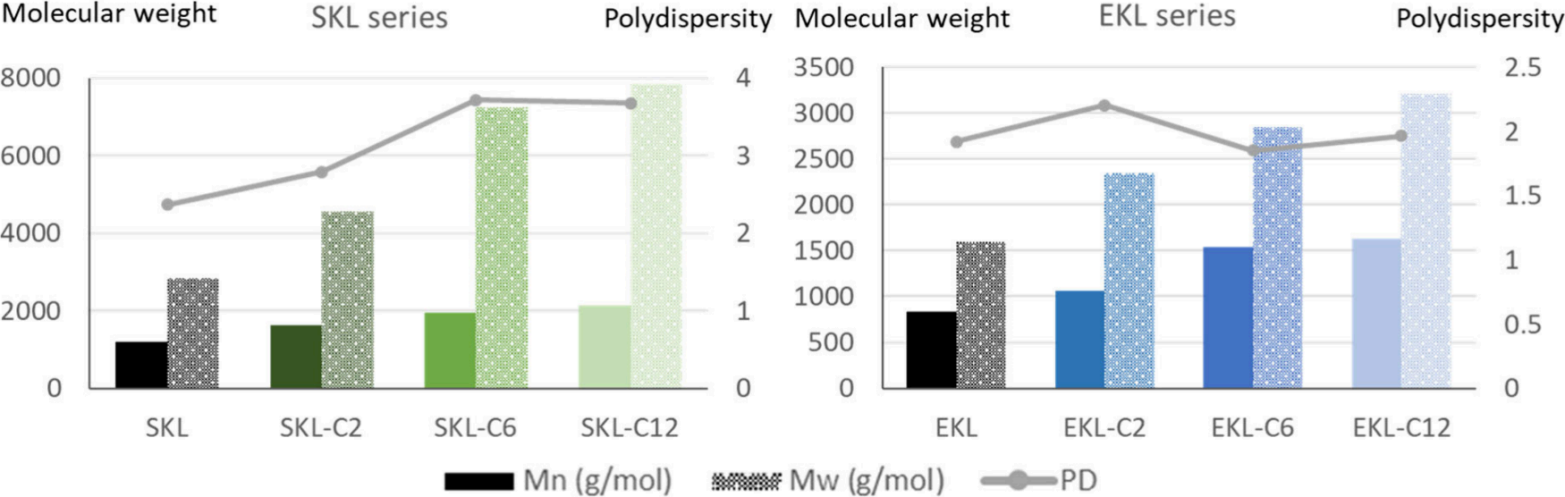
Molecular weight and polydispersity of unmodified and modified lignin by SEC.

**Table 2:** Weight-Related Changes in Lignin Samples

sample	overall *M*_n_ increase by SEC (w%)	estimated loaded ester groups by hydroxyl group consumption (w%)
SKL-C2	27	16
SKL-C6	39	18
SKL-C12	44	8
EKL-C2	27	18
EKL-C6	46	19
EKL-C12	49	9

The esterified lignin samples exhibited a pronounced increase in *M*_n_ and *M*_w_ when analyzed in THF by SEC, corresponding to the increasing length of the attached ester chains under otherwise identical conditions. This shift suggests an enhanced solvation and expansion of the esterified samples in THF, reflecting a greater hydrophobic character introduced by longer ester groups.^66^ The reduced polarity and hydrogen-bonding capacity of the esterified lignins as a result of hydroxyl group consumption likely suppress intra- and intermolecular associations in solution, allowing for more extended conformations with higher hydrodynamic volumes.^51^ The increased degree of number-average molecular weight was slightly higher in eucalyptus kraft lignin, likely due to its intrinsically lower molecular weight. As a result, the anchoring of an equivalent (or even lower) stoichiometric amount of ester chains induced more pronounced shifts in the hydrophobic/hydrophilic balance.

However, it is important to note that SEC results deviated from the estimation of ester group loading calculated from the actual extent of hydroxyl group conversion. Esterification alters not only the chemical structure but also the molecular flexibility and solvation properties of lignin, which in turn affect its hydrodynamic volume in a nonlinear manner. Consequently, even partial substitution of hydroxyl groups can lead to disproportionately large increases in the apparent molar mass. Moreover, steric effects and increased chain mobility from bulky or long acyl groups can further inflate the measured *M*_n_/*M*_w_, independent of the true degree of substitution.^67^

Consequently, the changes in the average molecular weight of samples were used solely to qualitatively reflect the overall changes in nonpolar solvent affinity (in other terms, hydrophobic characteristics) of lignin samples rather than to quantify the actual structural changes at the level of individual lignin macromolecules.

### Lignin Nanoparticle Self-Assembly

The self-assembly mechanisms of lignin nanoparticles through antisolvent precipitation could not be simply explained by the traditional phase separation theory of diblock copolymers.^68^ Kraft lignin derivatives differ fundamentally from typical amphiphilic polymers, with cross-linked macromolecules of a broad molecular weight distribution, formed by randomly linked phenyl-propanoid units. The Hydrophilic functional groups, primarily hydroxyl and carboxyl groups, are distributed marginally and randomly along the polymer backbone. The self-assembly process of lignin nanoparticles is considered primarily driven by intermolecular and intramolecular interactions within lignin macromolecules and solvent molecules, including polar forces (hydrogen bonding between functional groups) and nonpolar forces (π–π stacking of aromatic structures, hydrophobic interactions among lignin functional moieties and solvent molecules).^33^

The most hydrophobic regions of lignin consist mainly of condensed aromatic domains that are formed through extensive C–C linkages, contributing significantly to its overall hydrophobic character.^41^ There are regions with better hydrophilicity: (i) More sterically accessible domains, short end chains and functional groups such as phenolic/aliphatic hydroxyl groups, carboxyl groups, etc., attached at the molecular periphery of the rigid aromatic networks. (ii) Lower molecular weight fractions with limited aromatic condensation and relative abundance polar end groups. These domains are more exposed to the solvent and can readily form hydrogen bonds with water. Such balance between the hydrophobic/hydrophilic regions collectively influences the interactions of lignin in aqueous environments and therefore directs the self-assembly behaviors.^40,45^

During the addition of water as an antisolvent into the lignin acetone solution, more hydrophobic domains consisting mainly of condensed aromatic structures are likely to form local clusters due to the short-range hydrogen bonding from functional groups with aid of π–π stacking interactions among their aromatic groups; the more sterically accessible peripheral groups tend to rearrange to the outer surface of the primary colloidal clusters, due to their different affinity with water. These interactions are determined by the monolignol chemical structures, dominating the control over the primary nucleation of lignin colloidal spheres, ultimately influencing the eventual size of mature lignin nanoparticles.^43,69^ As acetone gradually diffuses out during the dialysis process, colloidal spheres undergo a maturation process driven by the progressive hydrophobic aggregation of relatively less hydrophobic and more sterically accessible domains, which meanwhile leads to a redistribution of chemical functionalities onto the surface.^57^ This process is particularly dependent on the hydrophilic–hydrophobic balance of lignin macromolecules. This reorganization of the nanoparticle surfaces optimizes their surface energy, finalizing nanoparticle growth into stable spherical structures.^70^ (The self-assembly process is shown in Figure 3.)

**Figure 3:**
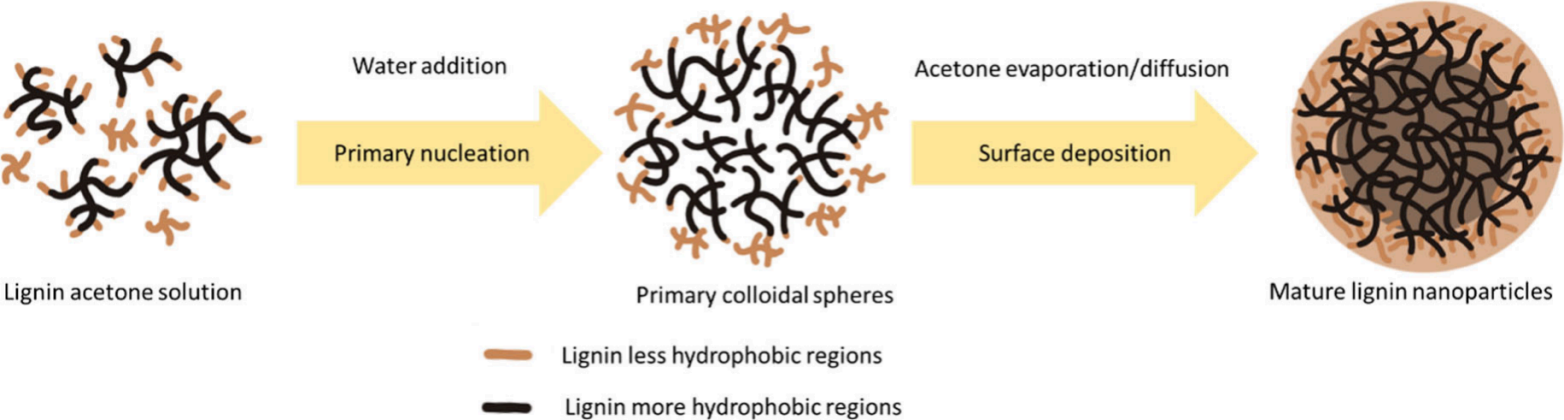
Proposed mechanisms of lignin nanoparticle self-assembly.

#### Evaluation of Lignin Nanoparticle Size and Morphology

The characteristic morphologies of lignin nanoparticles were observed by using TEM (Figure 4). The statistical distribution of nanoparticle hydrodynamic diameters was analyzed by DLS, with the results presented in Table 3 and Figure S5. The resulting lignin nanoparticles exhibited diverse size distributions, which were influenced by the chemical structure of the lignin precursors. A predominant fraction of the nanoparticles had diameters in the range of approximately 200 to 300 nm, while the overall hydrodynamic radius ranged from ∼ 100 to 800 nm.

**Figure 4:**
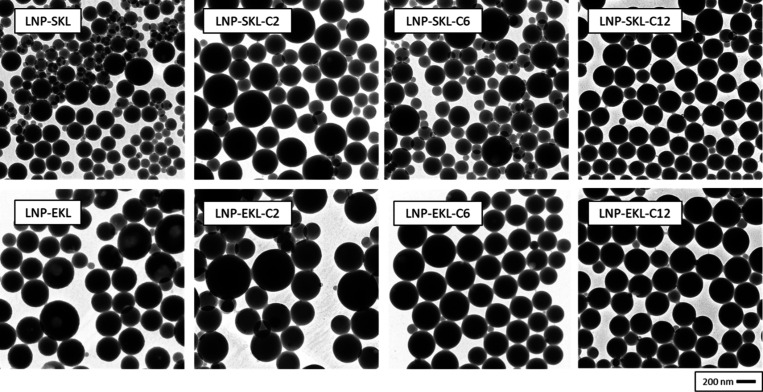
TEM micrographs of dispersed lignin nanoparticles (magnification: 130×, beam acceleration voltage: 100 kV, emission: 0.8 hA).

**Table 3:** Average Diameter and Polydispersity Index of Lignin Nanoparticle Water Suspension (0.1 mg/mL)

sample	hydrodynamic average (nm)	polydispersity index
LNP-SKL	175.8 ± 5.4	0.077 ± 0.005
LNP-SKL-C2	237.6 ± 9.3	0.083 ± 0.008
LNP-SKL-C6	223.5 ± 8.7	0.118 ± 0.009
LNP-SKL-C12	205.7 ± 4.5	0.065 ± 0.006
LNP-EKL	196.0 ± 6.3	0.122 ± 0.010
LNP-EKL-C2	294.2 ± 10.2	0.149 ± 0.009
LNP-EKL-C6	261.2 ± 7.3	0.117 ± 0.008
LNP-EKL-C12	246.9 ± 6.7	0.084 ± 0.006

The physiochemical properties of lignin, including monolignol precursor composition, degree of cross-linking, and functional group abundance, play a critical role in governing the average size of LNPs by determining the strength of the following inter- and intramolecular interactions.

#### Hydrogen Bonding

The lignin nanoparticles derived from unmodified kraft lignin (LNP-SKL and LNP-EKL) exhibited consistently smaller average sizes compared with their esterified counterparts. The unmodified lignin samples, rich in phenolic and aliphatic hydroxyl groups and carboxyl groups, readily form hydrogen bonds in the aqueous environment, thereby promoting the primary nucleation. These interactions were progressively weakened as the contents of hydroxyl groups decreased with increasing esterification intensity: the contents of hydroxyl groups in the esterified samples showed a negative correlation with the average sizes of the resulting nanoparticles, that the nanoparticles formed from C2-, C6- and C12-functionalized samples showed a decreasing trend of their average sizes. These findings underscore the pivotal role of hydrogen bonding, mediated by phenolic and aliphatic hydroxyl groups, in promoting efficient primary nucleation in aqueous environments.^71^ The highly condensed and cross-linked spruce kraft lignin bears higher contents of oxygenated functional groups, contributing to stronger hydrogen bond networks.^72^ This explained the generally larger particle sizes of LNPs derived from EKL in comparison to those from SKL.

Beyond the nucleation stage, hydrogen bonding also contributes to the compaction of lignin on nanoparticle surfaces during the maturation phase. Size difference and subtle packing density variations between lignin nanoparticles assembled from acetylated kraft lignin (LNP-SKL-C2 and LNP-EKL-C2) and their unmodified counterparts (LNP-SKL and LNP-EKL) suggest that hydrogen bonding played a significant role in promoting more regulated surface aggregation. In unmodified lignin samples, the abundance of hydroxyl groups enable strong intermolecular hydrogen bonding within adjacent less hydrophobic domains, leading to their more controlled deposition onto the nanoparticle surfaces, resulting in more compact particles.^73^ In contrast, acetylation of lignin disrupts the intrinsic hydrogen bonding network by removing the most significant amounts of hydroxyl groups without introduction of groups that provide sufficient hydrophobic interaction forces, in turn reducing particle surface compaction. The LNPs on the lignin samples functionalized with longer ester groups showed compact structures compared to those of the acetylated samples, which could also be explained by the preservation of hydroxyl groups in their structures that provided stronger hydrogen bonding to promote surface deposition.

#### π–π Stacking Forces

The generally larger sizes of LNPs derived from eucalyptus kraft lignin (LNP-EKL, LNP-EKL-C2, LNP-EKL-C6, and LNP-EKL-C12), compared to those from spruce kraft lignin (LNP-SKL, LNP-SKL-C2, LNP-SKL-C6, and LNP-SKL-C12), could be also ascribed to the differences in π–π stacking capacity decided by the intrinsic structural characteristics of the lignin precursors: Spruce kraft lignin derivatives are enriched in monosubstituted aromatic guaiacyl units that engage in intensive π–π stacking interactions. These interactions enhance the aggregation of the most hydrophobic lignin domains into compact colloidal nuclei during primary nucleation as well as the progressive deposition of other adjacent domains, thereby promoting the formation of smaller and denser LNPs.^57,74^

During the introduction of progressively longer ester groups to substitute hydroxyl functionalities in lignin, π–π stacking interactions would be disrupted due to increased steric hindrance.^75,76^ However, the extent to which this steric interference influences the size of LNPs remains unclear as the effects are confounded by variations in the degree of hydroxyl group preservation. It is not yet evident whether the influence of disrupted π–π stacking outweighs the impact of residual hydroxyl groups that continue to facilitate hydrogen bonding. Future work should aim to decouple these two variables by adjusting the conditions of C6- and C12-esterification to minimize differences in the hydroxyl group content. This would allow for a more precise evaluation of how the steric bulk of ester groups modulates π–π stacking interactions during nanoparticle assembly.

#### Hydrophobicity and Structural Rigidity

Hydrophobic interactions also play a crucial role in modulating the self-assembly and formation of lignin nanoparticles (LNPs). Lignin samples functionalized with ester groups of increasing chain lengths (from C2 to C6 and C12) yielded progressively smaller LNPs with subtle improvements in the size uniformity. This trend suggests that enhanced hydrophobic attraction facilitates more controlled hydrophobic aggregation during both nucleation and growth.^77^ The incorporation of longer alkyl chains significantly strengthens the repulsion of lignin toward water, driving the most hydrophobic domains to aggregate into compact colloidal spheres in order to minimize unfavorable interactions with the aqueous environment. This process also promotes the regulated deposition of less hydrophobic, low-molecular-weight lignin segments onto the particle surface, contributing to the formation of more morphologically uniform nanoparticles. Notably, these hydrophobic interactions act in concert with hydrogen bonding networks, collectively leading to a gradual decrease in the average LNP size as the length of the grafted hydrophobic chains increases.

Additionally, the differences in nanoparticle size and morphologies might be explained by variations in the monolignol rigidity and weight distributions of condensed aromatic domains. The higher average molecular weight and broader polydispersity of spruce kraft lignin derivatives facilitate rapid in situ nucleation of more hydrophobic domains, quickly depleting local supersaturated lignin concentrations and allowing less hydrophobic domains to participate in subsequent particle growth;^78,79^ In contrast, eucalyptus kraft lignin derivatives, which are less branched, lower in molecular weight and exhibit narrower polydispersity, exhibit higher and more homogeneous intramolecular adhesion tendencies, promoting cooperative participation of a larger population of lignin molecules in the secondary growth per nanoparticle, resulting in the formation of relevantly larger spheres.^40,80^

Interestingly, LNP-SKL-C6 did not exhibit noticeable improvements in nanoparticle uniformity control. It was probably due to the fact that the incorporation of hexanoyl ester groups via partial esterification was insufficient to fully override the decrease in hydrogen bonding forces and to regulate the uniform hydrophobic aggregation of LNPs. This suggested that an appropriate balance between hydrophobicity alteration, changes in aromatic stacking, and the hydrogen bonding network is essential for effectively producing LNPs with optimal size and morphology.

#### Evaluation of Lignin Nanoparticle Surface Properties

The surface characteristics of lignin nanoparticles, which govern their colloidal behaviors in suspensions and serve as an essential criteria for potential applications, were valued using ^1^H NMR, surface potential, and contact angle analysis on a hydrophilic surface.

#### Surface Functional Group Orientations

Characteristic signals of lignin aromatic substructures were identified by ^1^H NMR, as shown in Figures 5 and 6. These includes signals at 3.5–3.9 ppm (methoxy groups of syringyl units and guaiacyl units, overlapping with γ-protons of β–β structures),^81^ 6.7–7.0 ppm (aromatic protons), and 7.25–7.50 ppm (aromatic protons adjacent to aldehyde and ketones).^57^

**Figure 5:**
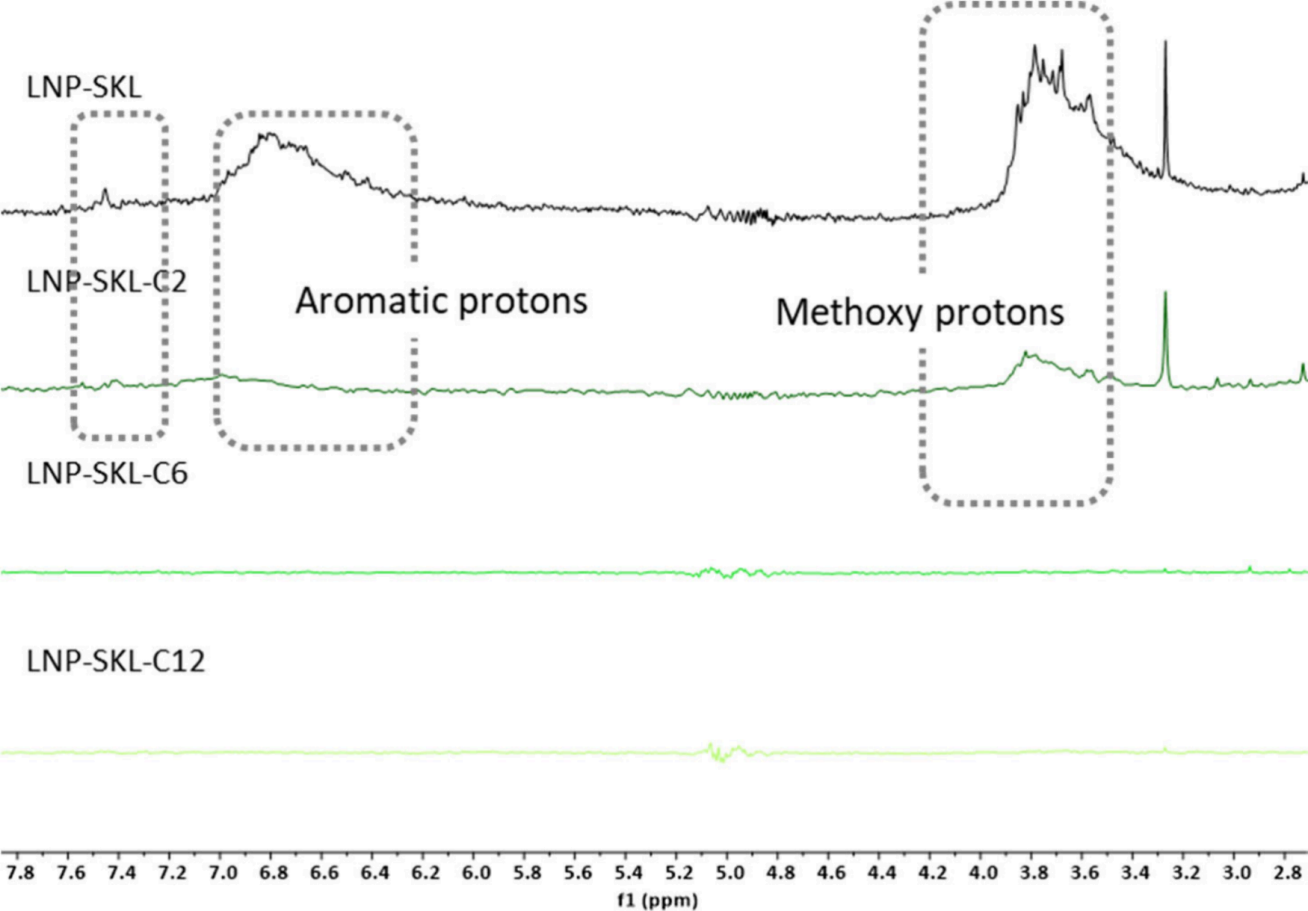
^1^H NMR spectra for lignin nanoparticle from SKL series in D_2_O suspensions.

**Figure 6:**
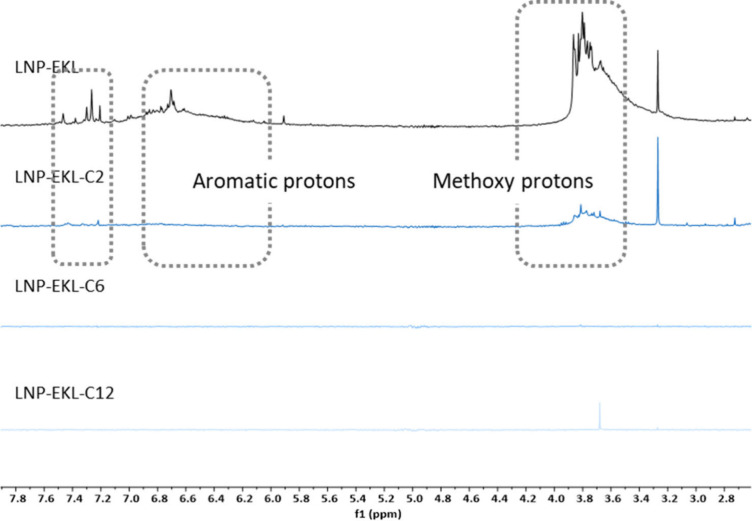
^1^H NMR spectra for lignin nanoparticle from EKL series as D_2_O suspensions.

The structural conformation and molecular arrangement of both modified and unmodified lignins during LNP formation were likely influenced by varying degrees of hydrophobic interactions and steric effects. The prominent presence of all of the mentioned peaks on the surfaces of LNP-SKL and LNP-EKL aligned with the abundance of intact phenolic structures in the unmodified lignin samples. The intensities of these signals decreased significantly in LNP-SKL-C2 and LNP-EKL-C2 samples, indicating a substantial reduction in the number of hydroxyl groups in the less hydrophobic and more sterically accessible domains on the surface. A small fraction of aromatic protons remained detectable, likely due to the acetyl groups of relatively lower steric hindrance that hardly mask signals from other functional moieties. These signals became undetectable in LNP-SKL-C6, LNP-SKL-C12, LNP-EKL-C6 and LNP-EKL-C12. It was plausible that, as stronger hydrophobic interactions increasingly dominated the surface aggregation process, such compact organization could hinder the orientation of aromatic structures from the most hydrophobic and sterically hindered domains toward the nanoparticle surface, potentially contributing to the suppression of the corresponding ^1^H NMR signals.

#### Surface Charge Distributions

The average zeta potentials of lignin nanoparticles in water suspensions measured by DLS are listed in Table 4, with the statistical distributions shown in Figure S6.

**Table 4:** Average Surface Potential of Lignin Nanoparticle Water Suspension (0.1 mg/mL)

sample	surface potential (mV)
LNP-SKL	–42.75 ± 0.23
LNP-SKL-C2	–20.55 ± 0.44
LNP-SKL-C6	–32.33 ± 0.78
LNP-SKL-C12	–26.94 ± 0.37
LNP-EKL	–42.76 ± 0.66
LNP-EKL-C2	–29.45 ± 0.30
LNP-EKL-C6	–35.72 ± 0.65
LNP-EKL-C12	–35.17 ± 0.55

All resultant lignin nanoparticles exhibited negative zeta potentials, with average values ranging from −20 to −40 mV, depending on the chemical structures of lignin. In neutral aqueous dispersions, the surface charges of lignin nanoparticles originate mainly from mildly deprotonated carboxylic acid groups, whereas the phenolic hydroxyl groups only gives out negative charges under high enough pH value.^82^ LNP-SKL and LNP-EKL, with intact hydroxyl moieties, showed the most negatively charged surfaces (−42.75 and −42.76 mV) among the nanoparticles assembled from esterified lignin. In contrast, LNP-SKL-C2 and LNP-EKL-C2, which underwent the most extensive esterification of hydroxyl groups, exhibited the least negative surface charges (−20.55 and −29.45 mV). Nanoparticles fabricated from C6- and C12-esterified lignin samples displayed intermediate surface charges. This may be attributed to the long ester chains in the surface layers that masked the signals of the unreacted hydroxyl groups.

#### Surface Hydrophilicity in Water Suspensions

The wetting property of resultant lignin nanoparticle solutions was compared by contact angle measurements, shown in Table 5.

**Table 5:** Contact Angles of Lignin Nanoparticles (2 mg/mL) Water Suspension on a Silicon Wafer

sample	contact angle (°)
pure water	35.5 ± 0.9
LNP-SKL	28.4 ± 0.9
LNP-SKL-C2	33.1 ± 1.3
LNP-SKL-C6	38.5 ± 1.5
LNP-SKL-C12	43.7 ± 0.9
LNP-EKL	28.5 ± 1.1
LNP-EKL-C2	36.3 ± 1.7
LNP-EKL-C6	38.7 ± 1.0
LNP-EKL-C12	43.7 ± 0.9

The LNP-SKL and LNP-EKL suspensions demonstrated good wettability, exhibiting significantly lower contact angles compared to water (which had a contact angle of 35.5° on the same silicon wafer). This behavior can be attributed to the abundance of hydrophilic hydroxyl groups and carboxyl groups on their surfaces, forming more hydrophilic surfaces and stabilizing the nanoparticle dispersions by electrostatic interactions. In contrast, the lignin nanoparticles assembled from the esterified samples generally displayed higher contact angles on the silicon wafer, likely due to the decrease of hydroxyl groups and carboxylic groups and the presence of sedimented aliphatic moieties forming more hydrophobic surfaces. Notably, LNP-SKL-C12 and LNP-EKL-C12 exhibited highest contact angles, suggesting that the compact packing of the longest ester chains on the surface likely masked the unesterified ionizable groups, lead to most hydrophobic surfaces.^82–84^ The results were in agreement with the ^1^H NMR spectra that almost no signals of hydrophilic moieties were detected in C6- and C12-esterified groups. Achieving an optimal balance in the chemical structure is therefore crucial for producing more uniform nanoparticles with improved wettability.

Given that lignins derived from spruce and eucalyptus, two sources with markedly different molecular conformations and structural features, were subjected to various esterification treatments and subsequently assembled into nanoparticles, a consistent trend was observed in how the esterification method influenced the resulting sizes, morphology, and surface properties of the lignin nanoparticles (LNPs). These observations suggest that beyond the inherent physicochemical characteristics of the lignin feedstocks, the modification-induced changes in noncovalent interactions, particularly hydrogen bonding, π–π stacking, and hydrophobic interactions, play a critical role in governing the self-assembly behaviors. In short, esterification not only alters the chemical structure of lignin but also modulates the balance of intermolecular forces that are central to the nanoparticle formation and performance.

## Conclusions

This study systematically investigated the influence of the hydrophobic moiety distribution on the self-assembly behaviors of lignin nanoparticles (LNPs). By introducing linear ester groups of varying chain lengths to kraft lignin derived from spruce and eucalyptus, this study demonstrated that esterification effectively altered the intra- and intermolecular interactions of lignin, thereby influencing the formation mechanisms and the resulting morphology and surface characteristics of the lignin nanoparticles.

This study is based on a refined theory of lignin nanoparticle solvent exchange self-assembly mechanisms, distinguishing two sequential stages: (i) primary nucleation, driven by aggregation of highly hydrophobic and sterically hindered lignin domains, and (ii) maturation, involving progressive surface deposition of less hydrophobic and more sterically accessible lignin domains. Both stages are modulated by the interactions and accessibility of lignin macromolecules within the binary solvent system. Our results showed that hydroxyl group substitution in lignin structures led to overall larger sizes of lignin nanoparticles, indicating the promotion of hydrogen bonding for tighter packing throughout the self-assembly process. In contrast, sufficient alteration of hydrophobicity through incorporation of long-chain ester groups resulted in the formation of regular and compact lignin nanoparticles with more hydrophobic surfaces than acetylated lignin nanoparticles, likely due to enhanced hydrophobic interactions facilitating the self-assembly with a well-regulated balance. This finding highlights hydrophobic interactions as an essential driving force governing self-assembly alongside hydrogen bonding and π–π stacking forces.

Collectively, this work provides a quantitative and insightful understanding of how specific hydrophobic features of lignin govern nanoparticle formation, leading to a more nuanced and experimentally grounded revision of the nucleation–maturation mechanism for LNPs that accounts for subtle but critical physicochemical variations. These insights establish a mechanistic framework for lignin nanoparticle production and offer a versatile strategy for the rational design of lignin-based nanomaterials with tailored properties for targeted applications.

## Supplementary Material



## Notes

FTIR signal assignments for lignin samples, HSQC spectra of lignin samples (with detected chemical structures demonstrated), HSQC signal assignments of lignin samples, relative linkage abundance of lignin samples, ^31^P NMR spectra of lignin samples, hydroxyl group abundance of lignin samples, SEC results of lignin samples, hydrophobic radius and zeta potential distributions of lignin nanoparticle samples (PDF)
