# Supplementary material for: Engineering of Industrial Kraft Lignin: The Role of Esterification Methods in Lignin Nanoparticle Self-Assembly
Source: Biomacromolecules. 2025 Aug 18;26(9):5727–39. doi: 10.1021/acs.biomac.5c00507 (PMC12421670; doi:10.1021/acs.biomac.5c00507)
Supplement: Supplementary file 1 [file bm5c00507_si_001.pdf]

Supporting Information for: Engineering of Industrial Kraft Lignin: the Role of Esterification Methods in Lignin Nanoparticle Self-assembly

Taoran Xu[1]†, Anastasia V. Riazanova[2], Pär A. Lindén[1,2], Gunnar Henriksson[1,2], L. Daniel Söderberg[1,2], Oihana Gordobil[3*] and Olena Sevastyanova[1,2*]

[1]*Wallenberg Wood Science Center, Department of Fiber and Polymer Technology, KTH Royal Institute of Technology, School of Chemistry, Biotechnology and Health, Teknikringen 56, Stockholm, 100 44, Sweden;*

[2]*Department of Fiber and Polymer Technology, School of Chemistry, Biotechnology and Health, Teknikringen 56, Stockholm, 100 44, Sweden;*

[3]*"Materials + Technologies" Research Group (GMT), Department of Chemical and Environmental Engineering, University of the Basque Country UPV/EHU, Faculty of Engineering of Gipuzkoa, Plaza Europa 1, Donostia-San Sebastián, 20018, Spain;*

*Email: olena@kth.se and oihana.gordobil@ehu.eus

Table S1 FTIR signal assignments of lignin samples

Sample	Peak Wavenumbers (cm^{-1})	Band structure
Common structure of lignin	Broad band around 3500	Hydroxyl groups O-H stretching vibration (aromatic/aliphatic)
	2942	Aromatic methoxy C-H stretching vibration
	2832	Aliphatic methyl C-H stretching vibration
	1705	Unconjugated C=O stretching vibration
	1680	Conjugated C=O stretching vibration
	1500-1515	Aromatic skeleton vibration
	1462	Aromatic ring C-H deformation vibration
	1215	Hydroxyl groups C-O stretching vibration (aromatic/aliphatic)
SKL	1270-1266	Guaiacyl ring vibration
	857	Guaiacyl ring C-H out of plane vibration
EKL	1325-1300	Syringyl + guaicyl condensed ring vibration
	1128	Syringyl ring vibration
	843	Syringyl C-H out of plane vibration
SKL-C2	2937	Aromatic methoxy C-H stretching vibration
	2832	Aliphatic methyl C-H stretching vibration
	1755	Aromatic acetoxy C=O stretching vibration
	1734	Aliphatic acetoxy C=O stretching vibration
	1365	Acetoxy C-H bending vibration
	1187	Aromatic acetoxy C-O stretching vibration
	1031	Aliphatic acetoxy C-O deformation vibration
EKL-C2	2934	Aromatic methoxy C-H stretching vibration
	2840	Aliphatic methyl C-H stretching vibration
	1762	Aromatic acetoxy C=O stretching vibration
	1738	Aliphatic acetoxy C=O stretching vibration
	1368	Hydroxyl O-H bending vibration
	1189	Aromatic acetoxy C-O stretching vibration

	1033	Aliphatic acetoxy C-O deformation vibration
SKL-C6	2958, 2929	Aromatic methyl C-H stretching vibration
	2861	Aliphatic methyl C-H stretching vibration
	1760	Aromatic carbonyl C=O stretching vibration
	1268, 1200, 1132, 1090	Carbonyl C-O stretching vibration
EKL-C6	2957, 2933	Aromatic methyl C-H stretching vibration
	2856	Aliphatic methyl C-H stretching vibration
	1754	Aromatic carbonyl C=O stretching vibration
	1331, 1269, 1130, 1090	Carbonyl C-O stretching vibration
SKL-C12	2954, 2921	Aromatic methyl C-H stretching vibration<br>romatic methyl C-H stretching vibration
	2852	Aliphatic methyl C-H stretching vibration
	1756	Aromatic carbonyl C=O stretching vibration
	1705	Aliphatic carbonyl C=O stretching vibration
	1331, 1210, 1130, 1110	Carbonyl C-O stretching vibration
EKL-C12	2954, 2923	Aromatic methyl C-H stretching vibration
	2852	Aliphatic methyl C-H stretching vibration
	1760	Aromatic carbonyl C=O stretching vibration
	1705	Aliphatic carbonyl C=O stretching vibration
	1265,1212, 1123, 1106	Carbonyl C-O stretching vibration

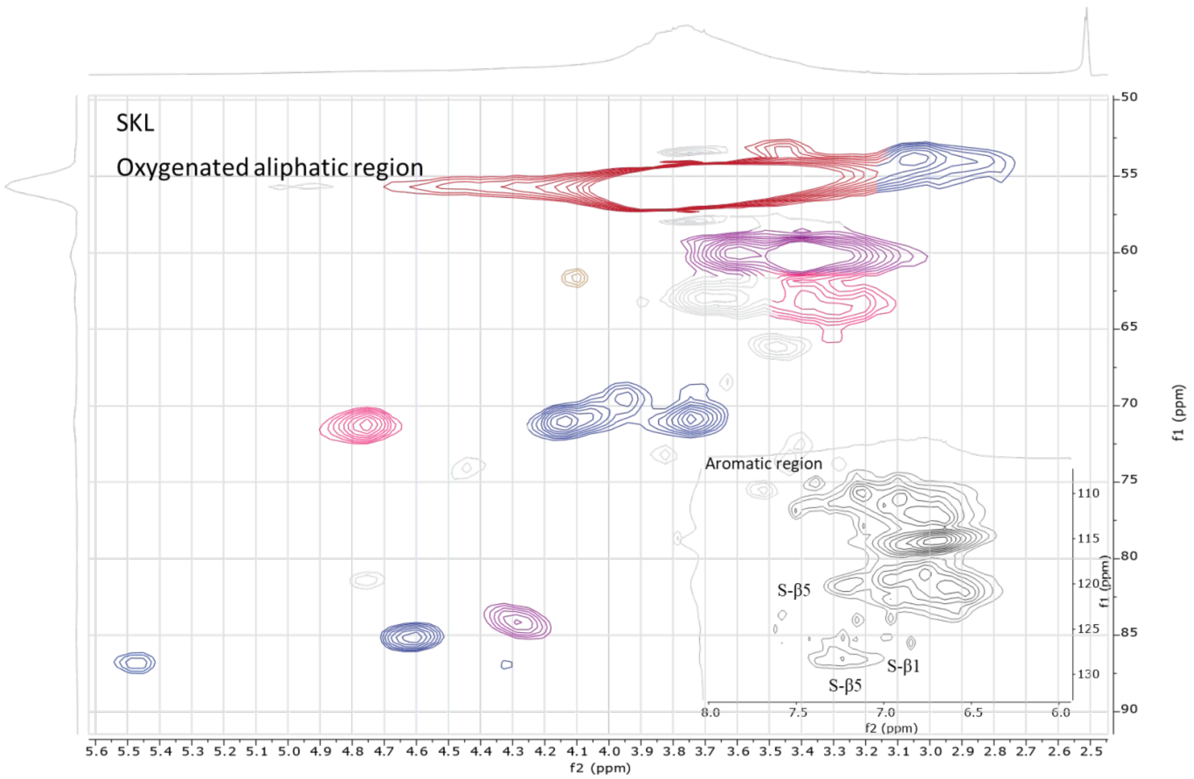
SKL
Oxygenated aliphatic region
Aromatic region
S-β5
S-β1
S-β5
f2 (ppm)
f1 (ppm)

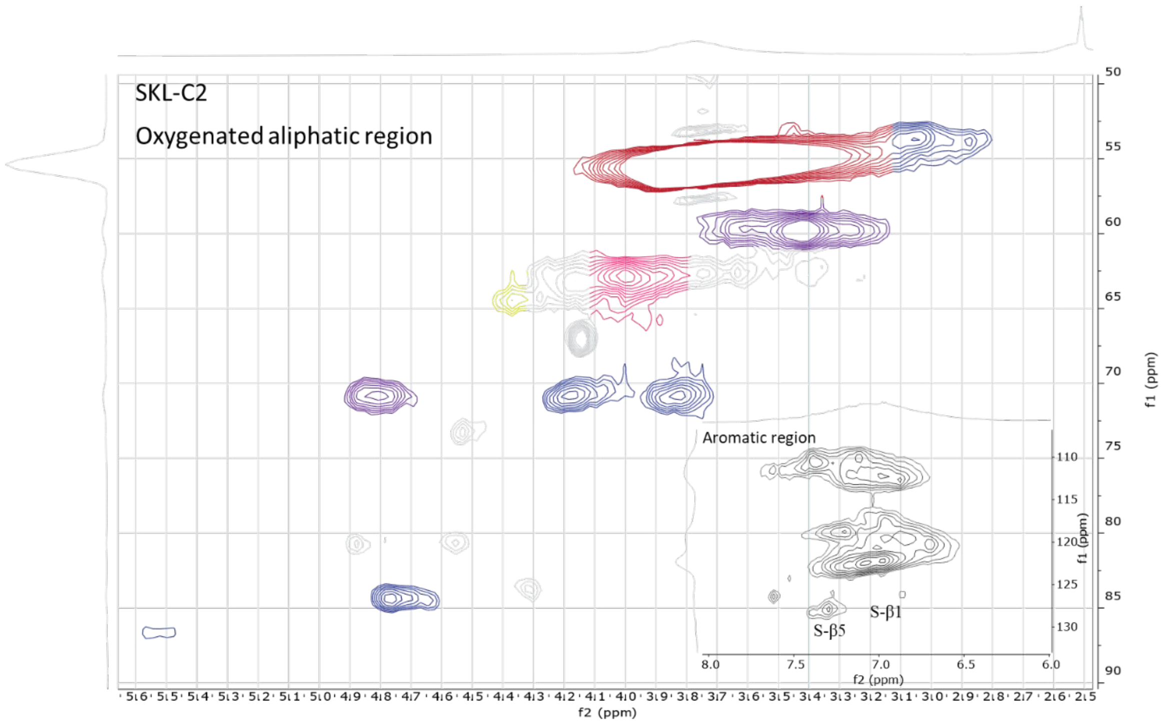
SKL-C2
Oxygenated aliphatic region
Aromatic region
S-β1
S-β5
f2 (ppm)
f1 (ppm)

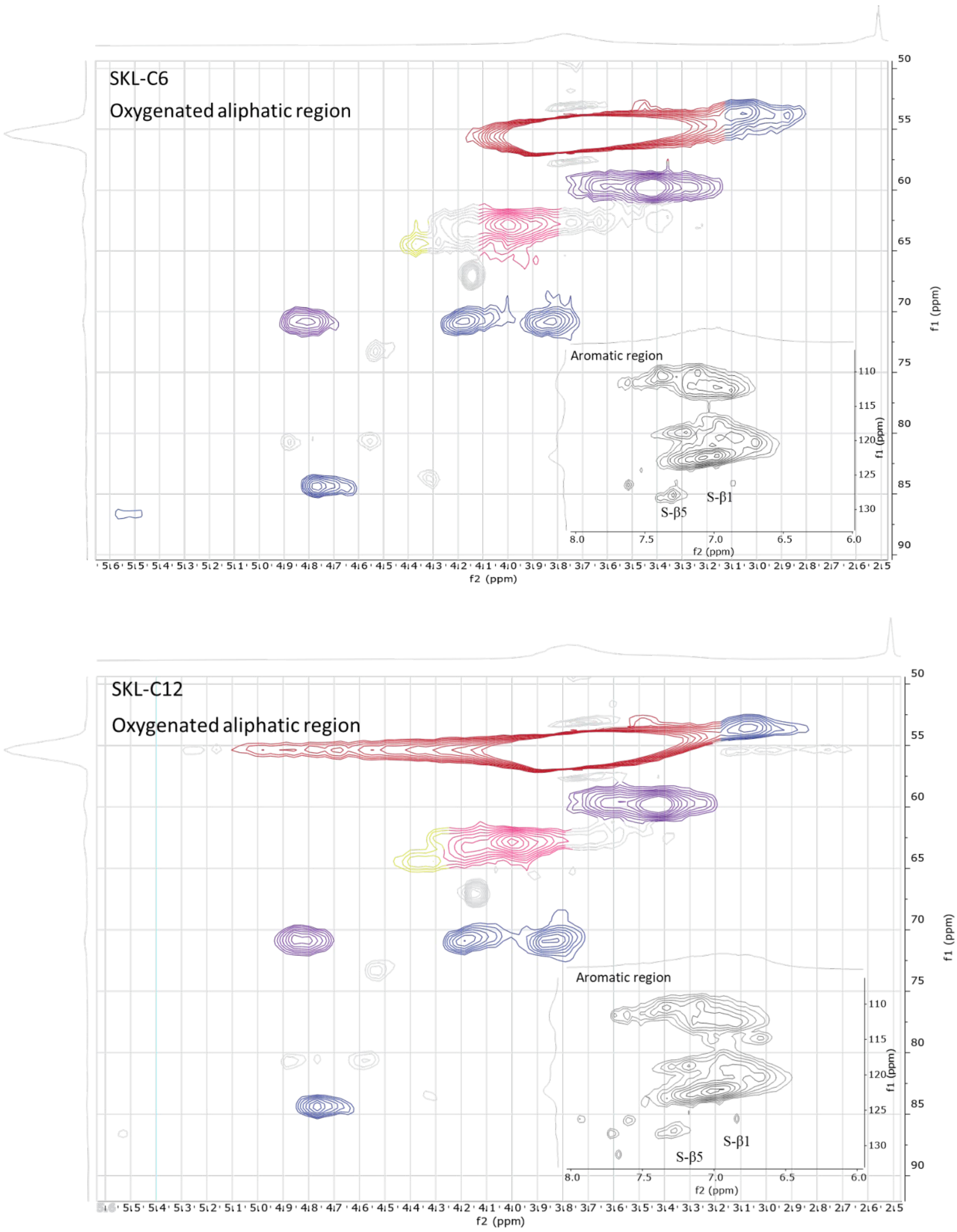

Figure S1 HSQC spectra of SKL derivatives

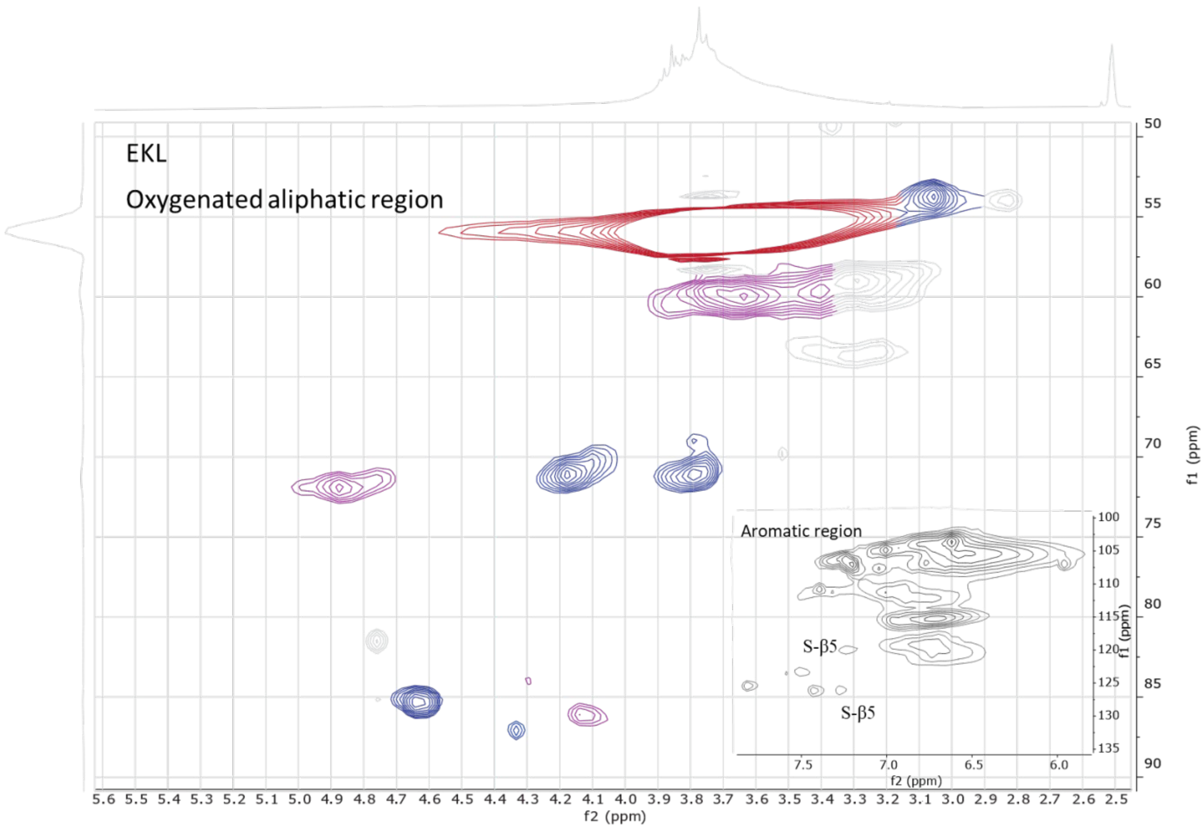
EKL
Oxygenated aliphatic region
Aromatic region
S-β5
S-β5
f2 (ppm)
f1 (ppm)

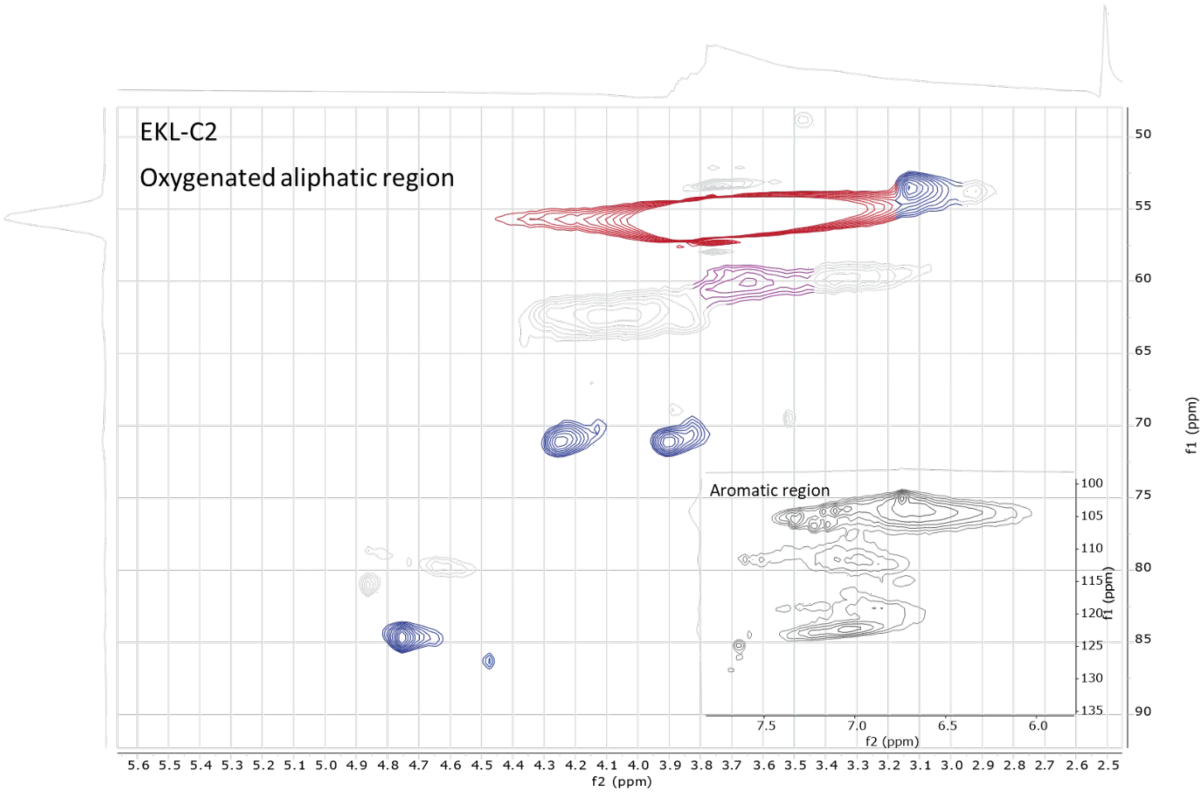
EKL-C2
Oxygenated aliphatic region
Aromatic region
f2 (ppm)
f1 (ppm)

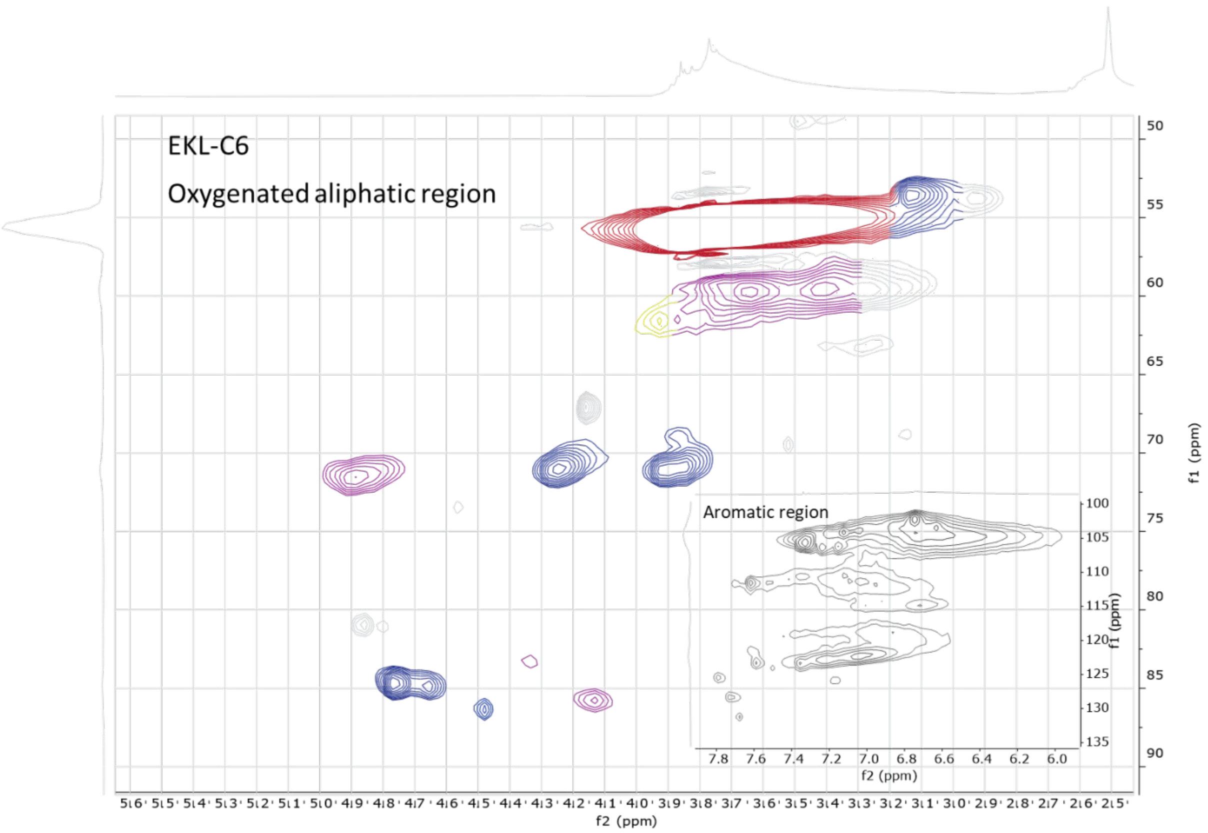

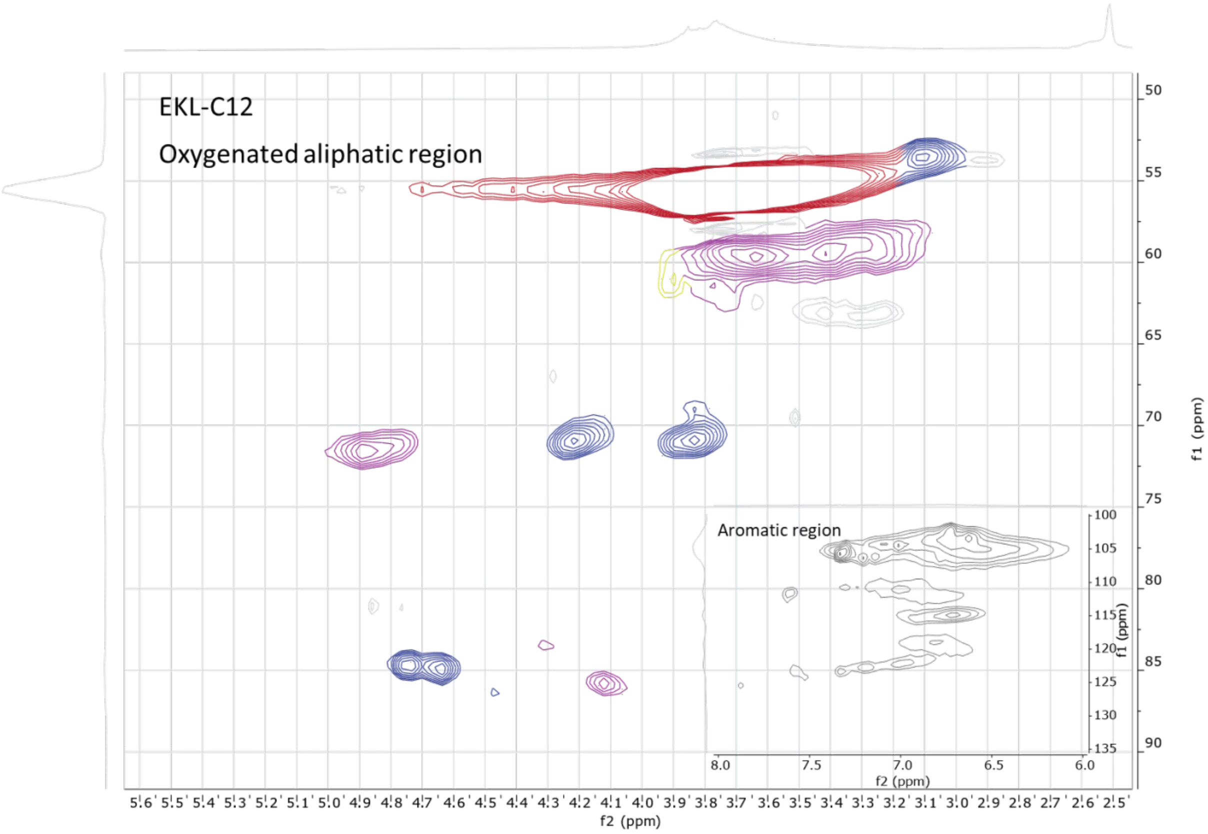

Figure S2 HSQC spectra of EKL derivatives

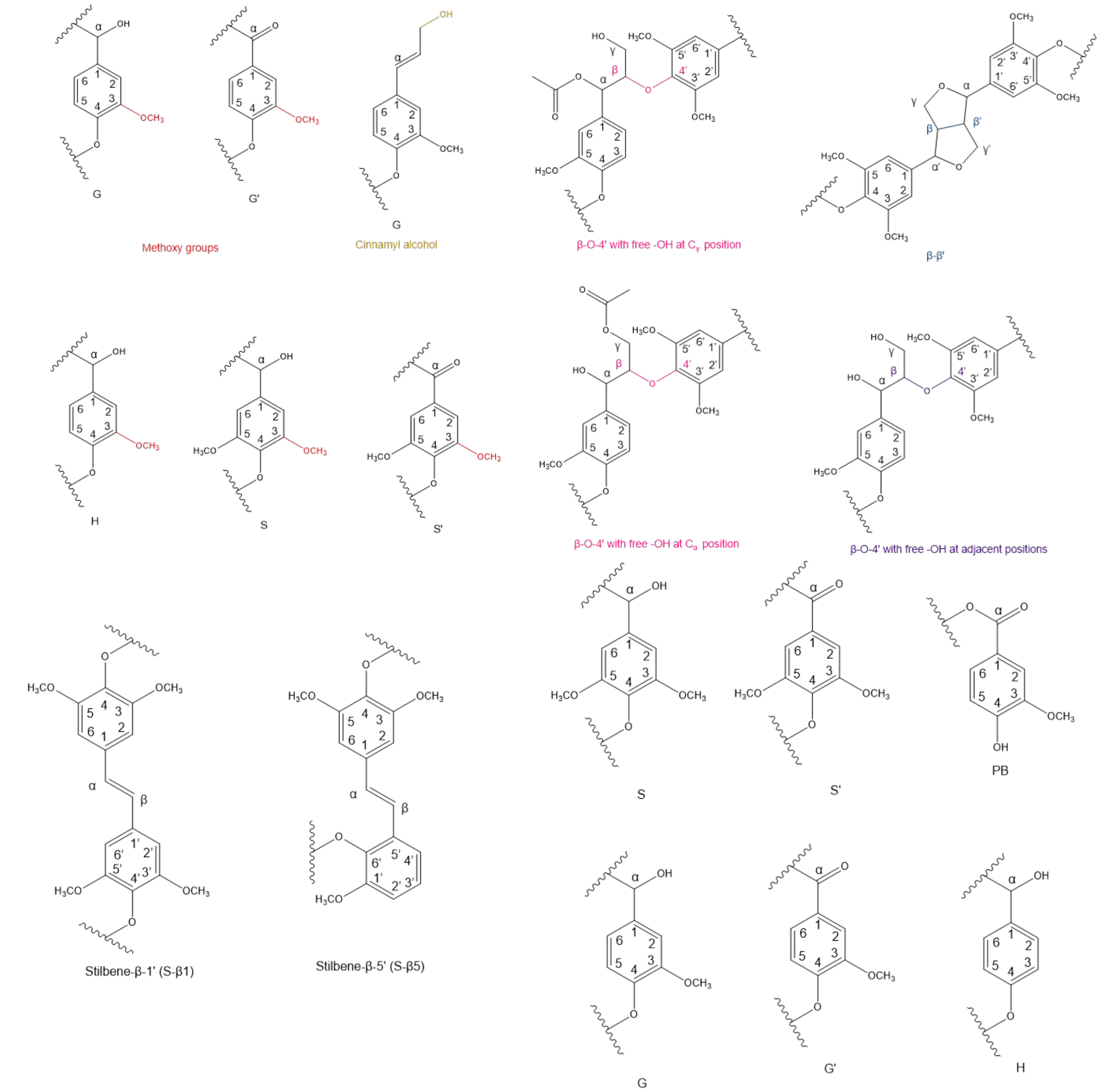

Figure S3 Chemical structures detected in HSQC

Table S2 HSQC signal assignments of SKL derivatives

Structures	δH/δC				Assignments
	SKL	SKL-C2	SKL-C6	SKL-C12	
**-OCH$_3$**	3.75/55.69	3.78/55.37	3.76/55.37	3.75/55.37	C-H in methoxy groups
**β-O-4**	3.60/59.90 (0.36) 3.40/60.22 (0.09)	-	3.42/59.90 (0.18)	3.42/59.90 (0.34)	C_λ -H_λ in A
	-	-	4.80/70.90 (0.04)	4.86/70.90 (0.04)	C_α-H_α in G (A)
	4.28/84.17 (0)	-	-	-	C_β-H_β in G (A)
**β-O-4 (acetylated)**	3.29/63.78 (0)	4.00/63.12 (0.73)	4.00/62.81 (0.13)	4.00/62.81 (0.22)	C_λ -H_λ in A’

	4.76/71.22<br>(0.02)	-	-	-	C_{α}-H_{α} in A′
**β-β′**	3.74/70.90<br>(0.02)<br>4.14/70.90<br>(0.02)	3.90/70.90<br>(0.01)<br>4.21/70.90<br>(0.01)	3.82/70.90<br>(0.03)<br>4.18/70.90<br>(0.01)	3.83/70.90<br>(0.12)<br>4.19/70.90<br>(0.1)	C_{λ} -H_{λ} in B
	4.61/85.14<br>(0.01)	4.78/84.49<br>(0.04)	4.77/84.49<br>(0.01)	4.77/84.49<br>(0.06)	C_{α}-H_{α} in B
	3.05/53.75<br>(0.07)	3.12/53.75<br>(0.05)	3.04/53.43<br>(0.04)<br>3.05/53.75<br>(0.01)	3.08/53.75<br>(0.11)	C_{β}-H_{β} in B
	5,47/86.75<br>(0)	5.54/86.75<br>(0)	5.56/86.75<br>(0)	5.53/86.43<br>(0.02)	C_{α}-H_{α} in C
	4.32/87.08<br>(0)	4.49/86.11<br>(0)	-	-	$C_{\alpha'}$-$H_{\alpha'}$ in D
**Cinnamyl acetate**	-	4.69/64.11	4.37/64.43	4.32/64.43	C_{λ} -H_{λ}
	-	-	-	-	C_{α}-H_{α}
**G unit**	6.91/110.70<br>6.99/111.5	6.88/112.64<br>6.98/112.31	6.84/112.64<br>6.95/112.31	6.85/112.64<br>6.96/112.31	C_2-C_2
	6.70/115.22		6.67/114.90	6.68/114.90	C_5-C_5
	6.76/118.78<br>6.96/119.43	6.85/119.43	6.68/120.40	6.68/120.40	C_6-C_6
**G Oxidized C_{α} α-ketone**	7.50/111.99	7.62/111.34	7.62/111.67	7.61/111.67	C_2-C_2/C_6-C_6
**PB**	-	-	7.67/131.40	7.67/131.08	C_2-C_2/C_6-C_6
	6.65/112.64<br>7.39/108.75	7.37/110.37	7.33/110.70	7.33/110.70	C_8-C_8
**H unit**	7.24/128.17	7.30/127.84	7.28/127.84	7.28/127.84	C_2-C_2/C_6-C_6
**Cinnamyl acetate**	6.84/126.55	6.86/126.23	6.85/126.23	6.84/126.23	C_{β}-H_{β}
**Stilbene-β-1′**	6.84/126.55	6.86/126.23	6.85/126.23	6.84/126.23	C_{α}-H_{α}
**Stilbene-β-5′**	7.24/128.17<br>(0.1)<br>7.21/120.8	7.30/127.84<br>(0.2)<br>-	7.28/127.84<br>(0.05)<br>-	7.28/127.84<br>(0.29)<br>-	C_{α}-H_{α}<br>C_{β}-H_{β}

Table S3 HSQC signal assignments of EKL derivatives

Structures	δH/δC				Assignments
	EKL	EKL-C2	EKL-C6	EKL-C12	
**-OCH_3**	3.75/56.02	3.76/55.69	3.76/55.69	3.74/55.69	C-H in methoxy groups
**β-O-4**	3.64/59.90<br>(0.19)	-	3.64/59.90<br>(0.15)	3.64/59.58<br>(0.22)	C_{λ} -H_{λ} in A
	3.40/59.58<br>(0.09)	3.65/60.22<br>(0.14)	3.41/59.58<br>(0.12)	3.42/59.58<br>(0.12)	C_{α}-H_{α} in G (A)
	4.88/71.87<br>(0.01)	-	4.89/71.55<br>(0.05)	4.86/71.55<br>(0.07)	C_{α}-H_{α} in S (A)
	4.30/83.84<br>()	-	4.34/83.19	4.32/83.52<br>()	C_{β}-H_{β} in G (A)
	4.14/86.11<br>()	-	4.13/85.78	4.12/85.78<br>(0.01)	C_{β}-H_{β} in S (A)
	6.00/74.78<br>()	5.92/73.81<br>(0.01)	-	-	C_{α}-H_{α} in A′

**β-β'**	4.18/71.22 (0.02)<br>3.79/71.22 (0.02)	4.25/71.22 (0.03)<br>3.90/71.22 (0.03)	4.24/70.90 (0.07)<br>3.90/71.22 (0.06)	4.21/70.90 (0.06)<br>3.83/70.90 (0.07)	C_{λ} -H_{λ} in B
	4.63/85.46 (0.02)	4.75/84.81 (0.02)	4.76/84.81 (0.05)<br>4.66/84.81 (0.01)	4.74/84.81 (0.02)<br>4,64/84.81 (0.02)	C_{α}-H_{α} in B
	3.06/53.5 (0.04)	3.13/53.43 (0.04)	3.13/53.43 (0.09)	3.10/53.43 (0.10)	C_{β}-H_{β} in B
	4.33/87.08 ()	4.48/86.43	4.48/86.43 ()	4.47/86.43	$C_{\alpha'}$-$H_{\alpha'}$ in D
**S unit**	6.62/103.58	6.74/102.28	6.75/102.28	6.63/103.25	C_2-C_2/C_6-C_6
**S oxidized C_{α}**	7.21/107.14<br>7.23/106.49	7.22/106.49	7.33/105.52<br>7.24/106.49	7.33/105.84<br>7.21/106.49	C_2-C_2/C_6-C_6
**G unit**	7.00/111.34	6.98/111.67	6.96/111.99	-	C_2-C_2
	6.71/115.22	-	6.72/114.90	6.71/114.90	C_5-C_5
	6.75/119.11	-	-	-	C_6-C_6
**Stilbene-β-5'**	7.32/127.20	-	-	-	C_{α}-H_{α}
	7.24/120.08	-	-	-	C_{β}-H_{β}

Table S4 Interlinkage relative abundance of SKL and EKL derivatives by HSQC

Sample	β-O-4'/ β-β'
**SKL**	2.73
**SKL-C2**	2.58
**SKL-C6**	2.62
**SKL-C12**	2.67
**EKL**	3.03
**EKL-C2**	2.92
**EKL-C6**	2.76
**EKL-C12**	2.88

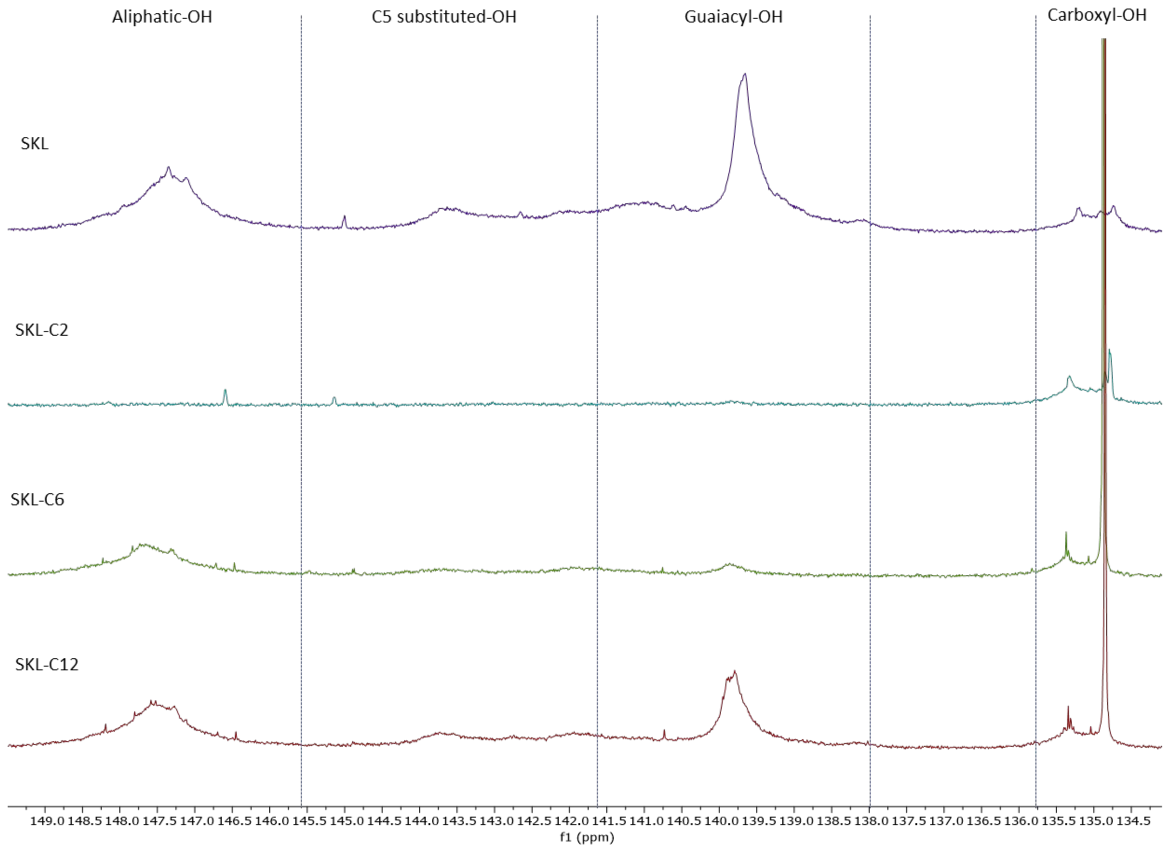

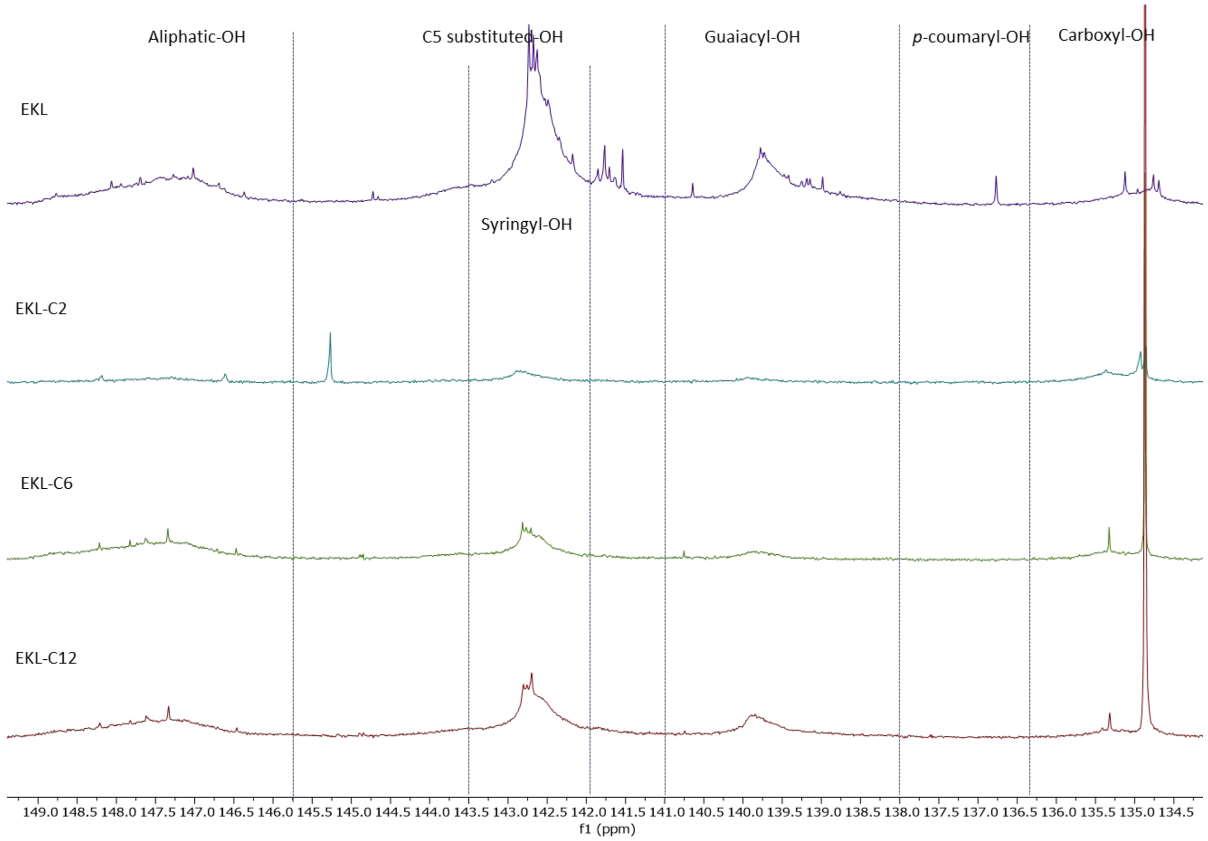

Figure S4 31P NMR spectra of SKL and EKL derivatives

Table S5 Quantification of hydroxyl groups in lignin

	**Aliphatic-OH (mmol/g)**	**Total phenolics (mmol/g)**	**Condensed phenolics (mmol/g)**		**Non-condensed Phenolics (mmol/g)**	
			**Condensed Guaiacyl-OH (mmol/g)**	**Syringyl -OH (mmol/g)**	**Non-condensed Guaiacyl-OH (mmol/g)**	***p*-hydroxyl -OH (mmol/g)**
**Shift**	149.1-145.1	144.7-137.3	144.7-141.1	143-142	140.6-138.8	138.7-137.3
**SKL**	1.83	3.64	1.47	/	2.01	0.15
**SKL-C2**	0.10	0.18	0.09	/	0.07	0.02
**SKL-C6**	0.85	0.64	0.42	/	0.19	0.03
**SKL-C12**	1.18	1.64	0.76	/	0.82	0.06
**EKL**	1.47	4.24	1.16	1.96	1.02	0.09
**EKL-C2**	0.14	0.23	0.08	0.08	0.06	0.01
**EKL-C6**	0.83	0.88	0.30	0.38	0.18	0.02
**EKL-C12**	0.85	2.00	0.42	0.68	0.87	0.03

* One issue to note was that residue carboxylic acids (converted from unreacted C6- and C12-esterification reagents) were observed in ^{31}P NMR analysis. These residues may influence the dissolution and aggregation behaviours of lignin during the subsequent processes of nanoparticle assembly. Such limitations of esterification methods have also been reported in other research[1] and the potential impacts of these residues remain to be further investigated. However, these linear chains with significantly lower molecular weights exhibit distinctly different structural behaviours in

organic solvent solutions, suggesting that they are unlikely to dominate the hydrophobic aggregation of lignin in acetone and water binary system[2].

Table S6 SEC results of lignin samples

	Mn (g/mol)	Mw (g/mol)	Polydispersity Index
**SKL**	1193	2824	2.3672
**SKL-C2**	1631	4546	2.7867
**SKL-C6**	1948	7230	3.7125
**SKL-C12**	2133	7841	3.6763
**EKL**	831	1596	1.9212
**EKL-C2**	1061	2341	2.2055
**EKL-C6**	1534	2840	1.8522
**EKL-C12**	1633	3206	1.9635

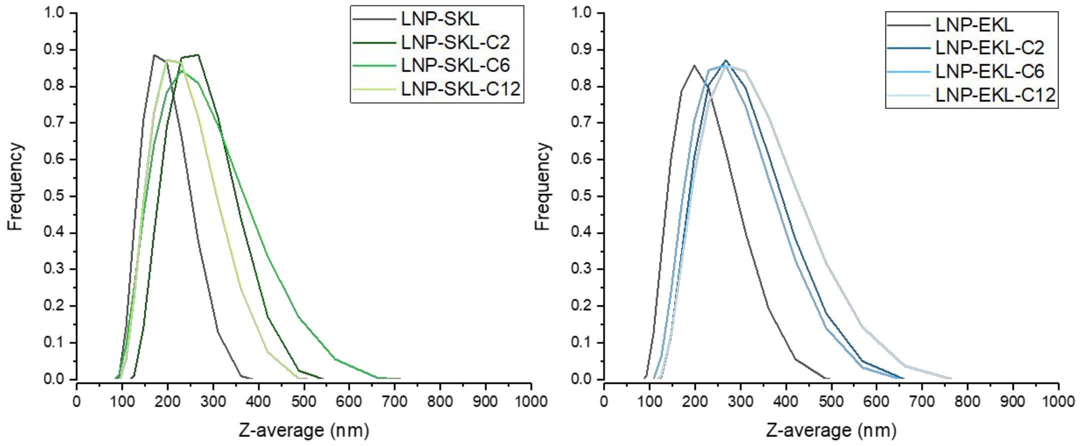

Figure S5 Hydrodynamic radius and distribution of lignin nanoparticles in 0.1 mg/mL suspension by DLS

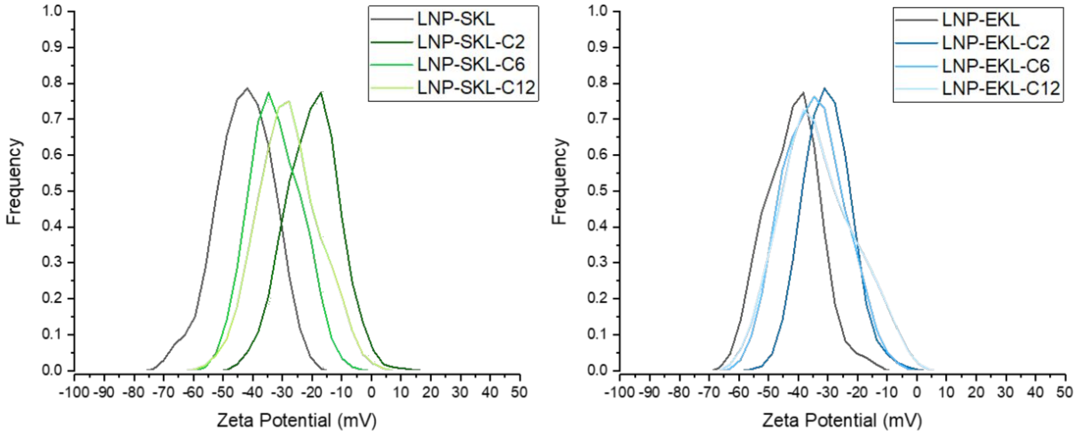

Figure S6 Surface zeta potential and distribution of lignin nanoparticles by DLS

(1) Koivu, K. A. Y.; Sadeghifar, H.; Nousiainen, P. A.; Argyropoulos, D. S.; Sipilä, J. Effect of Fatty Acid Esterification on the Thermal Properties of Softwood Kraft Lignin. *ACS Sustainable Chem. Eng.* **2016**, *4* (10), 5238–5247. https://doi.org/10.1021/acssuschemeng.6b01048.

(2) Zhang, X.; Zhang, J.; Yang, H.; He, C.; Ke, Y.; Singh, S.; Cheng, G. Determination of the Structures of Lignin Subunits and Nanoparticles in Solution by Small-Angle Neutron Scattering: Towards Improving Lignin Valorization. *ChemSusChem* **2022**, *15* (19), e202201230. https://doi.org/10.1002/cssc.202201230.
